# Impact of the Communities That HEAL Intervention on Buprenorphine-Waivered Practitioners and Buprenorphine Prescribing: A Prespecified Secondary Analysis of the HCS Randomized Clinical Trial

**DOI:** 10.1001/jamanetworkopen.2024.0132

**Published:** 2024-02-22

**Authors:** Thomas J. Stopka, Denise C. Babineau, Erin B. Gibson, Charles E. Knott, Debbie M. Cheng, Jennifer Villani, Jonathan M. Wai, Derek Blevins, James L. David, Dawn A. Goddard-Eckrich, Michelle R. Lofwall, Richard Massatti, Jolene DeFiore-Hyrmer, Michael S. Lyons, Laura C. Fanucchi, Daniel R. Harris, Jeffery Talbert, Lindsey Hammerslag, Devin Oller, Raymond R. Balise, Daniel J. Feaster, William Soares, Gary A. Zarkin, LaShawn Glasgow, Emmanuel Oga, John McCarthy, Lauren D’Costa, Rouba Chahine, Steve Gomori, Netrali Dalvi, Shikhar Shrestha, Chad Garner, Aimee Shadwick, Pamela Salsberry, Michael W. Konstan, Bridget Freisthler, John Winhusen, Nabila El-Bassel, Jeffrey H. Samet, Sharon L. Walsh

**Affiliations:** 1 Department of Public Health and Community Medicine, Tufts University School of Medicine, Boston, Massachusetts; 2 Research Triangle Institute, Research Triangle Park, North Carolina; 3 Department of Medicine, Boston Medical Center, Boston, Massachusetts; 4 Boston University School of Public Health, Boston, Massachusetts; 5 National Institute on Drug Abuse, National Institutes of Health, Bethesda, Maryland; 6 Department of Psychiatry, Columbia University; Division on Substance Use Disorders, New York State Psychiatric Institute, New York; 7 College of Medicine, University of Kentucky Center on Drug and Alcohol Research, Lexington; 8 Ohio Department of Mental Health and Addiction Services, Columbus; 9 State of Ohio Board of Pharmacy, Columbus; 10 College of Medicine, Ohio State University, Columbus; 11 College of Pharmacy, University of Kentucky, Lexington; 12 Department of Public Health Sciences, University of Miami, Miami, Florida; 13 UMass Chan Medical School–Baystate, Springfield, Massachusetts; 14 Office of Prescription Monitoring and Drug Control, Massachusetts Department of Public Health, Boston; 15 RecoveryOhio, Office of Ohio Governor Mike DeWine, Columbus; 16 Health Behavior and Health Promotion, Ohio State University, Columbus; 17 Case Western Reserve University School of Medicine, Cleveland, Ohio; 18 University of Cincinnati College of Medicine, Cincinnati, Ohio; 19 Clinical Addiction Research and Education Unit, Section of General Internal Medicine, Department of Medicine, Boston University Chobanian & Avedisian School of Medicine and Boston Medical Center, Boston, Massachusetts

## Abstract

**Importance:**

Buprenorphine significantly reduces opioid-related overdose mortality. From 2002 to 2022, the Drug Addiction Treatment Act of 2000 (DATA 2000) required qualified practitioners to receive a waiver from the Drug Enforcement Agency to prescribe buprenorphine for treatment of opioid use disorder. During this period, waiver uptake among practitioners was modest; subsequent changes need to be examined.

**Objective:**

To determine whether the Communities That HEAL (CTH) intervention increased the rate of practitioners with DATA 2000 waivers and buprenorphine prescribing.

**Design, Setting, and Participants:**

This prespecified secondary analysis of the HEALing Communities Study, a multisite, 2-arm, parallel, community-level, cluster randomized, open, wait-list–controlled comparison clinical trial was designed to assess the effectiveness of the CTH intervention and was conducted between January 1, 2020, to December 31, 2023, in 67 communities in Kentucky, Massachusetts, New York, and Ohio, accounting for approximately 8.2 million adults. The participants in this trial were communities consisting of counties (n = 48) and municipalities (n = 19). Trial arm randomization was conducted using a covariate constrained randomization procedure stratified by state. Each state was balanced by community characteristics including urban/rural classification, fatal opioid overdose rate, and community population. Thirty-four communities were randomized to the intervention and 33 to wait-list control arms. Data analysis was conducted between March 20 and September 29, 2023, with a focus on the comparison period from July 1, 2021, to June 30, 2022. Data analysis was re-run on June 6, 2024, using a revised set of data that was locked on May 31, 2024.

**Intervention:**

Waiver trainings and other educational trainings were offered or supported by the HEALing Communities Study research sites in each state to help build practitioner capacity.

**Main Outcomes and Measures:**

The rate of practitioners with a DATA 2000 waiver (overall, and stratified by 30-, 100-, and 275-patient limits) per 100 000 adult residents aged 18 years or older during July 1, 2021, to June 30, 2022, were compared between the intervention and wait-list control communities. The rate of buprenorphine prescribing among those waivered practitioners was also compared between the intervention and wait-list control communities. Intention-to-treat and per-protocol analyses were performed.

**Results:**

A total of 8 211 506 individuals aged 18 years or older were residents of the 67 communities studied. There was no evidence of an effect of the CTH intervention on the adjusted rate of practitioners with a DATA 2000 waiver (adjusted relative rate [ARR], 1.04; 95% CI, 0.94-1.14) or the adjusted rate of practitioners with a DATA 2000 waiver who actively prescribed buprenorphine (ARR, 1.00; 95% CI, 0.91-1.10).

**Conclusions and Relevance:**

In this randomized clinical trial, the CTH intervention was not associated with increases in the rate of practitioners with a DATA 2000 waiver or buprenorphine prescribing among those waivered practitioners. Supporting practitioners to prescribe buprenorphine remains a critical yet challenging step in the continuum of care to treat opioid use disorder.

**Trial Registration:**

ClinicalTrials.gov Identifier: NCT04111939

## Key Points

**Question:**

Did the Communities That HEAL intervention increase the rate of practitioners with a Drug Addiction Treatment Act of 2000 waiver and prescribing of buprenorphine?

**Findings:**

In this secondary analysis of a randomized clinical trial including 67 communities accounting for approximately 8.2 million adults, there were no significant differences in the rate of waivered practitioners or active prescribing of buprenorphine between the intervention and wait-list control communities. There was no evidence of an effect of the intervention on the adjusted rate of practitioners with a DATA 2000 waiver or the adjusted rate of practitioners with a DATA 2000 waiver who actively prescribed buprenorphine.

**Meaning:**

The findings of this study suggest that the intervention was not beneficial regarding the rate of practitioners with a DATA 2000 waiver or prescribing of buprenorphine.

## Teaser

This secondary analysis of the HEALing Communities Study (HCS) randomized clinical trial investigates the effect of the Communities That HEAL intervention on rates of buprenorphine prescribers with waivers from the Drug Enforcement Agency and the rates of buprenorphine prescribing.

## Introduction

Increases in fatal opioid-related overdoses have been ubiquitous in the US,^1,2,3^ contributing to nearly 83 000 deaths in 2022.^4^ Recent data indicate that increasing fatal opioid-related overdose disparities are disproportionally impacting racial and ethnic minoritized groups.^5^

Medications for opioid use disorder (MOUD), particularly the opioid agonist methadone and partial agonist buprenorphine, are highly effective at reducing the negative health consequences associated with nontherapeutic opioid use, including fatal opioid-related overdoses.^6,7,8,9^ Although US federal regulations currently limit outpatient methadone dispensing for opioid use disorder (OUD) to licensed opioid treatment programs, buprenorphine may be prescribed from any clinical setting and dispensed from pharmacies.^10,11^ Despite proven efficacy,^12^ MOUD is underused. A recent study found that 22% of US residents with OUD in 2021 had received MOUD treatment in the last year.^13^ Racial and ethnic inequities in access to MOUD are prevalent.^14,15,16^

From 2002 to 2022, US physicians were required to obtain a Drug Addiction Treatment Act of 2000 (DATA 2000) waiver to prescribe buprenorphine for the treatment of OUD, with federal legislation in 2018 extending the waiver to nurse practitioners and physician assistants.^10^ In April 2021, the requirement to obtain a waiver to treat up to 30 individuals was eliminated.^11,17^ In December 2022, the fiscal 2023 congressional spending bill removed all buprenorphine waiver requirements and per-prescriber patient limits (MAT Act).^18^ Despite the easing of regulatory restrictions, buprenorphine prescribing has been insufficient,^19^ and interventions aimed at increasing prescribing are needed.

The HEALing Communities Study (HCS)^20^ is a randomized community-based trial evaluating the impact of implementing an integrated set of evidence-based practices from the Communities That HEAL (CTH) intervention framework delivered across health care, behavioral health, criminal legal system, and other community-based settings. The goal of HCS is to reduce opioid-related overdose deaths in highly affected communities in Kentucky, Massachusetts, New York, and Ohio. The CTH framework includes interventions to increase buprenorphine prescribing. Many communities aimed to increase the number of waivered practitioners or encourage inactive prescribers to begin prescribing. The objectives of the present study were to determine whether the CTH intervention resulted in a higher rate of practitioners with DATA 2000 waivers and more buprenorphine prescribing among waivered practitioners.

## Methods

### Trial Design

This study is a prespecified secondary analysis of the HCS, a multisite, 2-arm, parallel, community-level, cluster randomized, open, wait-list–controlled comparison clinical trial designed to assess the effectiveness of the CTH intervention on factors that could favorably impact the opioid overdose epidemic, including increasing uptake of buprenorphine waivers among practitioners and active buprenorphine prescribing. The trial protocol is given in Supplement 1. This report followed the Consolidated Standards of Reporting Trials (CONSORT) reporting guideline for randomized clinical trials. The study protocol was approved by Advarra Inc, the HCS single institutional review board. It was granted a waiver of consent and a full waiver of Health Insurance Portability and Accountability Act authorization for secondary data analysis. The data safety monitoring board was an independent group charged with monitoring the safety of participants and data quality.

### Study Population

Sixty-seven communities in Kentucky, Massachusetts, New York, and Ohio accounting for 8 211 506 residents aged 18 years or older were included. Communities consist of counties (n = 48) and municipalities (n = 19).

### Study Procedures and Intervention

Details have been published on the HCS vision,^21^ goals,^22^ study protocol,^23^ Opioid-overdose Reduction Continuum of Care Approach^24^ (the compendium of evidence-based practices [EBPs] and resources designed to facilitate EBP implementation), community engagement to support the adoption and sustainability of EBPs,^25^ health communication campaigns to increase demand for EBPs and reduce stigma,^26^ and community dashboards to support data-driven adoption of EBPs.^27^ Several strategies implemented through the CTH supported increasing the rate of DATA 2000–waivered practitioners and their buprenorphine prescribing rates.^28^ Strategies included DATA 2000 waiver trainings to expand the number of waivered clinicians, including free online training offerings, as well as Beyond the Waiver trainings, providing waivered prescribers with technical assistance and ancillary support services to encourage increased prescribing rates and the launching of new programs. The CTH intervention focuses on the implementation of EBPs within 3 categories expected to decrease fatal opioid overdoses: (1) overdose education and naloxone distribution, (2) delivery of MOUD, and (3) safer opioid prescribing and dispensing.^22,28^

### Randomization

The HCS Data Coordinating Center (DCC) randomized communities to trial arm using a covariate constrained randomization procedure^29,30,31^ stratified by state. Within each state, intervention and wait-list control arms were balanced by baseline community characteristics, including urban/rural classification, fatal opioid overdose rate, and community population. Communities were randomized to intervention (n = 34) or wait-list control (n = 33) conditions.^23^

### Timeline

The baseline period was 2019. Intervention communities received the CTH intervention from January 1, 2020, to June 30, 2022; the comparison period between intervention and wait-list control communities was July 1, 2021, to June 30, 2022. Analyses were performed between March 20 and September 29, 2023, with a focus on the comparison period from July 1, 2021, to June 30, 2022. On March 5, 2024, the HCS DCC for this study was informed by research partners about a potential issue with the source data sent from the states for this analysis. The HCS Data Capture Work Group reviewed stratified data for both intervention and wait-list control arms and determined that the measure specification for DATA 2000 waivered practitioners who prescribed buprenorphine was not consistently applied across the 4 states, at source, before these data were received at the HCS DCC. This new information was not known to the authors or the HCS DCC until after the data analysis was performed and after the study was published. The state public health data partners were contacted in March 2024 requesting that they consistently apply the measure specification and re-pull the data. Complete data were received by the 4 research sites and sent to the DCC on May 28, 2024. After a thorough quality control review, the data were locked by the HCS DCC on May 31, 2024. Analyses were re-run by the HCS DCC on June 6, 2024, in accordance with the original statistical analysis plan using the updated data transmitted to the HCS DCC, and revised results were provided.

### Study Outcomes and Data Sources

The primary outcome for the present study was the rate of practitioners with a DATA 2000 waiver overall, with secondary measures examining the 30-, 100-, and 275-patient limits, per 100 000 individuals aged 18 years or older in each community during the comparison period. The secondary outcome was the rate of waivered practitioners who actively prescribed buprenorphine in each community during the comparison period. Practitioners were defined as active if they wrote 1 or more prescriptions for buprenorphine filled by a resident of their state. For the comparison period, clinicians were counted only once in each community. If they changed their prescribing level during the period, they were counted at the highest level. If, for instance, someone in July 2021 had a 30-patient limit and then transitioned to the 100-patient limit in May 2022, they were counted once as having a 100-patient limit for the comparison period. In doing so, we avoided double counting clinicians by prescription level status.

Drug Enforcement Agency (DEA) data contained records of clinicians licensed in HCS states.^10^ Prescription Drug Monitoring Program (PDMP) data^32^ provided dispensing records for buprenorphine. The numerator for waivered practitioners was the sum of those listed within the DEA database as registered buprenorphine-waivered practitioners within a participating HCS community during the comparison period. The denominator was the number of individuals aged 18 years or older in HCS communities during the most recent calendar year available from the US Census Bureau’s American Community Survey and the National Center for Health Statistics Bridged-Race Resident Population Estimates.^33,34^ To determine the rate of actively prescribing waivered practitioners, the numerator was calculated using linked DEA-waivered practitioners data and PDMP data, and the denominator was calculated using all waivered practitioners registered in participating HCS communities. To protect confidentiality, PDMP data were subject to suppression rules (1-4 for Massachusetts and Ohio, 1-5 in Kentucky and New York), according to state policies. Data use agreements allowed Kentucky and New York to provide values in the suppressed range for statistical analyses only, while this was disallowed for Ohio and Massachusetts. We also recorded the number of buprenorphine DATA 2000 Waiver trainings and Beyond the Waiver trainings, and others, by state.

### Sample Size

The HCS was designed to have high power (>99%) to detect a 40% reduction in the primary outcome, fatal opioid overdoses.^23^ The study was not specifically powered for the current analyses.

### Statistical Analysis

The statistical analysis plan is presented in Supplement 2. For each outcome, descriptive statistics were calculated to summarize the number of events, population, and rate by intervention and subgroup (state, urban/rural classification, and patient limit). Sixty-seven communities were included in the intention-to-treat (ITT) population. One community randomized to the CTH intervention withdrew after randomization; thus, 66 of 67 communities were included in the per-protocol population (Figure). Each community-level outcome in the ITT population was analyzed using a negative binomial regression with robust, empirical, sandwich SE estimates and small-sample adjustments.^35,36^ Each model included the following community-level fixed effects: trial arm (intervention or wait-list control), state, urban/rural classification, baseline fatal opioid overdose rate, and the outcome rate (or the natural log of the rate, depending on the outcome) during the baseline period. The natural log of the community population size during the comparison period was also included as an offset term for models of the number of practitioners with a DATA 2000 waiver. For models of the number of waivered practitioners who actively prescribed buprenorphine, the offset was the natural log of the number of practitioners with a DATA 2000 waiver in the community. Outcomes suppressed in Massachusetts and Ohio communities were estimated through multiple imputation to ensure that each community was included in each analysis. An estimate of the adjusted rate and 95% CI of each outcome by intervention was reported (adjusting for state, urban/rural classification, the baseline fatal opioid overdose rate, and the baseline rate or natural log of the baseline rate of the outcome), as well as the adjusted relative rate (ARR) of the outcome comparing intervention and wait-list control.

**Figure.**
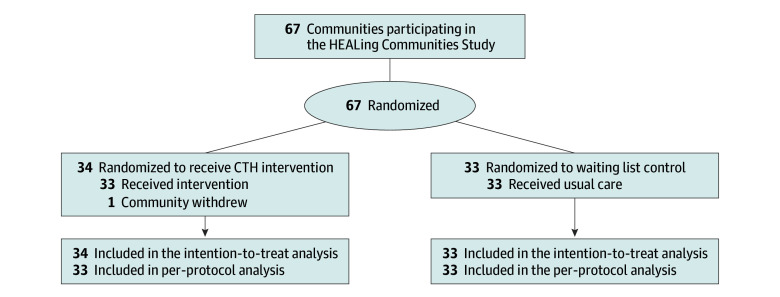
Flow Diagram of the HEALing Communities Study in Kentucky, Massachusetts, New York, and Ohio

Prespecified exploratory subgroup analyses using the ITT and per-protocol populations were also performed by including an interaction term between trial arm and a stratification variable representing the subgroup of interest (state, urban/rural classification, and patient limit). Suppressed values were excluded from these analyses. To control the false discovery rate (FDR), the Benjamini-Hochberg procedure^37^ was applied to all *P* values. All analyses were based on a 2-sided significance level of .05 and conducted using SAS, version 9.4 (SAS Institute Inc).

## Results

### Baseline Characteristics

Baseline characteristics of communities randomized to intervention (n = 4.4 million adult residents) and wait-list control (n = 3.8 million) are summarized in Table 1. Intervention communities (n = 34, of which 15 were rural), consisted of 8 communities each from Kentucky, Massachusetts, and New York, and 10 communities from Ohio. Wait-list control communities (n = 33, 14 rural) consisted of 8 communities each from Kentucky, Massachusetts, and New York, and 9 communities from Ohio. The mean (SD) baseline fatal opioid overdose rate was similar between intervention (38.2 [22.8] per 100 000 residents) and wait-list control (37.1 [20.3] per 100 000 residents) communities.

**Table 1.** Baseline Demographic Characteristics of 67 Communities Participating in the HEALing Communities Study, 2019

Characteristic	Intervention	Wait-list control	Overall
No. of randomized communities, No.	34	33	67
State, No. (%)			
Kentucky	8 (23.5)	8 (24.2)	16 (23.9)
Massachusetts	8 (23.5)	8 (24.2)	16 (23.9)
New York	8 (23.5)	8 (24.2)	16 (23.9)
Ohio	10 (29.4)	9 (27.3)	19 (28.4)
Urban/rural classification, No. (%)			
Urban	19 (55.9)	19 (57.6)	38 (56.7)
Rural	15 (44.1)	14 (42.4)	29 (43.3)
Population aged ≥18 y^a^			
Total, No.	4 439 170	3 772 336	8 211 506
Mean (SD)	130 563.8 (200 008.0)	114 313.2 (201 417.3)	122 559.8 (199 385.0)
Rate of fatal opioid overdoses, mean (SD)^b^	38.2 (22.8)	37.1 (20.3)	37.7 (21.4)
**Rate of practitioners with a DATA 2000 waiver** ^b^			
Mean (SD)	64.6 (50.4)	64.1 (64.9)	64.4 (57.6)
State, mean (SD)			
Kentucky	72.9 (38.5)	26.2 (15.8)	49.5 (37.3)
Massachusetts	96.3 (78.9)	142.4 (88.7)	119.4 (84.5)
New York	49.4 (14.3)	52.0 (18.0)	50.7 (15.8)
Ohio	44.9 (39.5)	39.1 (32.4)	42.1 (35.4)
Urban/rural classification, mean (SD)			
Urban	76.1 (57.0)	76.4 (74.4)	76.2 (65.4)
Rural	50.1 (37.3)	47.6 (47.0)	48.8 (41.5)
**Rate of practitioners with a DATA 2000 waiver with a 30-patient limit** ^b^			
Mean (SD)	41.8 (42.5)	45.0 (50.5)	43.4 (46.3)
State, mean (SD)			
Kentucky	30.2 (19.5)	15.5 (10.0)	22.9 (16.8)
Massachusetts	73.1 (72.9)	104.0 (73.4)	88.5 (72.5)
New York	34.6 (12.9)	35.3 (15.3)	35.0 (13.7)
Ohio	31.9 (30.3)	27.4 (20.3)	29.7 (25.4)
Urban/rural classification, mean (SD)			
Urban	51.5 (51.3)	54.5 (60.2)	53.0 (55.2)
Rural	29.6 (24.2)	32.1 (30.9)	30.8 (27.2)
**Rate of practitioners with a DATA 2000 waiver with a 100-patient limit** ^b^			
Mean (SD)	14.0 (10.3)	12.5 (13.2)	13.3 (11.8)
State, mean (SD)			
Kentucky	24.0 (12.5)	4.8 (7.6)	14.4 (14.1)
Massachusetts	14.5 (9.5)	26.1 (18.0)	20.3 (15.2)
New York	10.8 (2.7)	11.3 (6.1)	11.1 (4.5)
Ohio	8.2 (7.4)	8.4 (8.1)	8.3 (7.5)
Urban/rural classification, mean (SD)			
Urban	15.6 (10.4)	14.7 (13.1)	15.2 (11.7)
Rural	12.0 (10.1)	9.5 (13.2)	10.8 (11.5)
**Rate of practitioners with a DATA 2000 waiver with a 275-patient limit** ^b^			
Mean (SD)	8.8 (9.9)	6.6 (6.8)	7.7 (8.6)
State, mean (SD)			
Kentucky	18.6 (13.5)	5.9 (8.4)	12.2 (12.7)
Massachusetts	8.7 (9.8)	12.3 (6.1)	10.5 (8.1)
New York	3.9 (2.8)	5.4 (3.7)	4.7 (3.3)
Ohio	4.8 (3.9)	3.4 (5.9)	4.1 (4.8)
Urban/rural classification, mean (SD)			
Urban	9.0 (8.9)	7.1 (7.5)	8.1 (8.2)
Rural	8.5 (11.5)	6.0 (6.0)	7.3 (9.2)
**Rate of practitioners with a DATA 2000 waiver who actively prescribe buprenorphine products that are FDA-approved for OUD to state residents** ^c^			
Mean (SD)	53.2 (16.1)	54.7 (18.3)	53.9 (17.1)
Communities with missing data due to suppression, No. (%)	3 (8.8)	5 (15.2)	8 (11.9)
State			
Kentucky, mean (SD)	63.2 (8.6)	57.6 (20.8)	60.4 (15.6)
Communities with missing data due to suppression, No. (%)	0	0	0
Massachusetts, mean (SD)	52.4 (12.8)	46.4 (23.8)	49.2 (19.1)
Communities with missing data due to suppression, No. (%)	1 (12.5)	0	1 (6.3)
New York, mean (SD)	57.0 (14.8)	62.4 (10.8)	59.7 (12.8)
Communities with missing data due to suppression, No. (%)	0	0	0
Ohio, mean (SD)	40.0 (18.8)	49.9 (3.2)	43.3 (15.8)
Communities with missing data due to suppression, No. (%)	2 (20.0)	5 (55.6)	7 (36.8)
Urban/rural classification			
Urban, mean (SD)	52.6 (16.3)	52.6 (9.3)	52.6 (13.3)
Communities with missing data due to suppression, No. (%)	0	2 (10.5)	2 (5.3)
Rural, mean (SD)	54.1 (16.6)	57.9 (27.3)	55.9 (21.9)
Communities with missing data due to suppression, No. (%)	3 (20.0)	3 (21.4)	6 (20.7)
Patient limit			
30, mean (SD)	39.9 (18.7)	36.8 (23.9)	38.3 (21.3)
Communities with missing data due to suppression, No. (%)	7 (20.6)	6 (18.2)	13 (19.4)
100, mean (SD)	76.2 (16.7)	77.1 (28.2)	76.6 (22.3)
Communities with missing data due to suppression, No. (%)	8 (23.5)	12 (36.4)	20 (29.9)
275, mean (SD)	92.6 (8.5)	94.5 (8.3)	93.5 (8.4)
Communities with missing data due to suppression, No. (%)	12 (35.3)	13 (39.4)	25 (37.3)

Abbreviations: DATA, Drug Addiction Treatment Act; FDA, Food and Drug Administration; OUD, opioid use disorder.

^a^ Population information was drawn from the US Census Bureau for communities that represent counties (n = 48 of 67)^33^ and communities that represent units smaller than counties (n = 19 of 67).^34^

^b^ Rate was calculated using the ratio of the number of events that occurred during the baseline period within a community to the number of individuals aged 18 years or older during the baseline period within a community multiplied by 100 000.

^c^ Rate was calculated using the ratio of the number of practitioners with a DATA 2000 waiver who actively prescribed buprenorphine products that are FDA-approved for OUD to state residents during the baseline period within a community to the number of practitioners with a DATA 2000 waiver during the baseline period within a community multiplied by 100.

### Rate of Practitioners With a DATA 2000 Waiver

During the baseline period, the mean (SD) rate of practitioners with a DATA 2000 waiver per 100 000 residents was 64.6 (50.4) among intervention and 64.1 (64.9) among wait-list control communities included in the ITT population (Table 1). The mean (SD) rate of practitioners with a DATA 2000 waiver with a 30-patient limit per 100 000 residents was 41.8 (42.5); with a 100-patient limit per 100 000 residents, 14.0 (10.3); and with a 275-patient limit per 100 000 residents, 8.8 (9.9) among intervention communities. For wait-list control communities, the mean (SD) rate of practitioners with a DATA 2000 waiver with a 30-patient limit per 100 000 residents was 45.0 (50.5); with a 100-patient limit per 100 000 residents, 12.5 (13.2); and with a 275-patient limit per 100 000 residents, 6.6 (6.8) (eFigure in Supplement 3). Descriptive statistics for these rates are also provided by state and urban/rural classification in Table 1.

During the comparison period, the mean (SD) rate of practitioners with a DATA 2000 waiver per 100 000 residents was 96.8 (72.4) among intervention and 91.1 (89.8) among wait-list control communities (eFigure in Supplement 3). The mean (SD) rate of practitioners among intervention communities with a DATA 2000 waiver with a 30-patient limit per 100 000 residents was 58.4 (57.0); with a 100-patient limit per 100 000 residents, 24.6 (16.4); and with a 275-patient limit per 100 000 residents, 13.9 (13.8). For wait-list control communities, the mean (SD) rate of practitioners with a DATA 2000 waiver with a 30-patient limit per 100 000 residents was 59.0 (66.0); with a 100-patient limit per 100 000 residents, 22.7 (21.8); and with a 275-patient limit per 100 000 residents, 9.4 (7.3) (eFigure in Supplement 3). Descriptive statistics of the population size during the comparison period used to calculate these rates are provided in eTable 1 in Supplement 3. Descriptive statistics for these rates are also provided by state and urban/rural classification in Table 2.

**Table 2.** Number, Rate, and Relative Rate Between Intervention and Wait-List Control Communities of Practitioners With a DATA 2000 Waiver in the ITT Population

Outcome and group	Intervention			Wait-list control			Relative rate^a^
	Communities, No.^b^	No. of practitioners with a DATA 2000 waiver, mean (SD)^c^	Rate, mean (SD)^d^	Communities, No.^b^	No. of practitioners with a DATA 2000 waiver, mean (SD)^c^	Rate, mean (SD)^d^	
**Practitioners with a DATA 2000 waiver, No.**							
Overall	34	122.9 (184.5)	96.8 (72.4)	33	108.2 (219.6)	91.1 (89.8)	1.06
State							
Kentucky	8	90.4 (135.1)	106.9 (48.2)	8	60.6 (139.2)	45.4 (18.5)	2.35
Massachusetts	8	69.3 (62.1)	142.9 (123.6)	8	102.0 (93.3)	190.4 (131.3)	0.75
New York	8	113.1 (123.8)	76.3 (16.5)	8	98.1 (111.3)	78.1 (28.6)	0.98
Ohio	10	199.5 (292.6)	68.2 (45.0)	9	165.1 (389.0)	54.9 (50.7)	1.24
Urban/rural classification							
Urban	19	195.1 (222.5)	115.4 (84.8)	19	166.8 (276.5)	110.0 (104.5)	1.05
Rural	15	31.3 (22.9)	73.3 (45.1)	14	28.8 (31.0)	65.3 (59.0)	1.12
**Practitioners with a DATA 2000 waiver with a 30-patient limit, No.**							
Overall	34	81.1 (131.0)	58.4 (57.0)	33	71.8 (148.7)	59.0 (66.0)	0.99
State							
Kentucky	8	45.1 (81.5)	43.4 (27.1)	8	34.9 (82.1)	23.7 (12.4)	1.83
Massachusetts	8	47.4 (49.2)	94.4 (105.6)	8	69.9 (67.3)	127.9 (102.1)	0.74
New York	8	79.4 (86.2)	53.7 (9.2)	8	66.4 (78.8)	50.9 (20.5)	1.05
Ohio	10	138.1 (210.5)	45.2 (32.5)	9	111.1 (264.6)	36.3 (34.0)	1.25
Urban/rural classification							
Urban	19	131.2 (158.9)	73.4 (69.6)	19	111.6 (187.4)	72.5 (79.8)	1.01
Rural	15	17.6 (16.0)	39.3 (27.2)	14	17.7 (19.0)	40.7 (35.8)	0.96
**Practitioners with a DATA 2000 waiver with a 100-patient limit, No.**							
Overall	34	28.0 (39.6)	24.6 (16.4)	33	24.6 (45.1)	22.7 (21.8)	1.08
State							
Kentucky	8	24.3 (32.9)	31.9 (12.6)	8	15.9 (35.7)	12.9 (12.7)	2.47
Massachusetts	8	16.8 (13.4)	37.3 (22.4)	8	24.1 (20.9)	46.3 (29.5)	0.81
New York	8	25.0 (27.9)	17.6 (6.7)	8	22.6 (24.9)	19.9 (10.9)	0.88
Ohio	10	42.5 (61.5)	14.1 (8.8)	9	34.4 (76.8)	12.8 (11.5)	1.10
Urban/rural classification							
Urban	19	43.7 (47.5)	28.1 (18.5)	19	37.4 (56.1)	28.0 (22.0)	1.00
Rural	15	8.2 (5.7)	20.1 (12.5)	14	7.2 (9.3)	15.5 (20.1)	1.30
**Practitioners with a DATA 2000 waiver with a 275-patient limit, No.**							
Overall	34	13.8 (18.0)	13.9 (13.8)	33	11.9 (26.8)	9.4 (7.3)	1.48
State							
Kentucky	8	21.0 (21.2)	31.6 (15.6)	8	9.9 (21.5)	8.8 (6.7)	3.59
Massachusetts	8	5.1 (3.8)	11.2 (10.0)	8	8.0 (7.1)	16.1 (8.3)	0.70
New York	8	8.8 (10.6)	5.0 (2.9)	8	9.1 (8.5)	7.2 (3.3)	0.69
Ohio	10	18.9 (24.1)	8.9 (6.2)	9	19.6 (47.6)	5.8 (6.1)	1.54
Urban/rural classification							
Urban	19	20.3 (21.8)	13.9 (12.5)	19	17.8 (34.3)	9.6 (6.2)	1.45
Rural	15	5.5 (5.0)	13.9 (15.7)	14	3.9 (4.1)	9.1 (8.8)	1.53

Abbreviations: DATA, Drug Addiction Treatment Act; ITT, intention-to-treat.

^a^ Relative rate in the specified group was calculated as the ratio of the mean rate of providers with a DATA 2000 waiver in the specified intervention group during the comparison period to the mean rate of practitioners with a DATA 2000 waiver in the specified wait-list control group during the comparison period.

^b^ Number of communities randomized in the specified group that are included. Communities in the specified group that have suppressed event and/or population data are not included.

^c^ Mean (SD) of the number of practitioners with a DATA 2000 waiver in the specified group during the comparison period.

^d^ Mean (SD) of the rate of practitioners with a DATA 2000 waiver in the specified group during the comparison period. Rate in the specified group within a community was calculated as the ratio of the number of practitioners with a DATA 2000 waiver in the specified group within a community to the number of individuals aged 18 years or older in the specified group within a community multiplied by 100 000.

The absolute number of practitioners with a DATA 2000 waiver and the raw rate per 100 000 residents among intervention and wait-list control communities overall and for practitioners with a 30-, 100-, and 275-patient limit are included in eTable 2 in Supplement 3. Descriptive statistics for these rates are also provided by state and urban/rural classification.

In adjusted analyses of the ITT population, there was no evidence of a difference in the rate of practitioners with a DATA 2000 waiver between intervention and wait-list control communities (ARR, 1.04; 95% CI, 0.94-1.14) (Table 3). There was also no evidence of a difference between intervention and wait-list control communities in the rate of practitioners with a DATA 2000 waiver with a 30-patient (ARR, 1.05: 95% CI, 0.92-1.19), 100-patient (ARR, 1.15; 95% CI, 0.92-1.44), and 275-patient (ARR, 1.03; 95% CI, 0.80-1.33) limit. Furthermore, there was no evidence to suggest that the effect of the CTH intervention compared with wait-list control on the rate of practitioners with a DATA 2000 waiver differed by state or urban/rural classification (FDR-adjusted *P* value for interaction = 0.96 for each). Analyses using the per-protocol population also indicated nonsignificant findings.

**Table 3.** Adjusted Rates of Each Outcome Within Intervention and Wait-List Control Communities and Adjusted Relative Rate of Each Outcome

Outcome	Adjusted rate (95% CI)		Adjusted relative rate (95% CI)	*P* value
	Intervention	Wait-list control		
Practitioners with a DATA 2000 waiver, No.^a^^,^^b^	69.45 (64.41-74.89)	67.09 (61.95-72.65)	1.04 (0.94-1.14)	.46
Practitioners with a DATA 2000 waiver with a 30-patient limit, No.^a^^,^^c^	41.04 (36.69-45.92)	39.10 (35.22-43.41)	1.05 (0.92-1.19)	.45
Practitioners with a DATA 2000 waiver with a 100-patient limit, No.^d^^,^^e^	21.04 (18.29-24.22)	18.28 (15.10-22.12)	1.15 (0.92-1.44)	.21
Practitioners with a DATA 2000 waiver with a 275-patient limit, No.^d^^,^^f^	10.24 (8.68-12.08)	9.89 (8.14-12.02)	1.03 (0.80-1.33)	.79
Practitioners with a DATA 2000 waiver who actively prescribe buprenorphine products that are FDA-approved for OUD to state residents, No.^g^^,^^h^	46.48 (42.82-50.45)	46.57 (43.18-50.21)	1.00 (0.91-1.10)	.97

Abbreviations: DATA, Drug Addiction Treatment Act; FDA, Food and Drug Administration; OUD, opioid use disorder.

^a^ Results obtained from a negative binomial model adjusting for intervention, state (Kentucky, Massachusetts, New York, Ohio), urban/rural classification, baseline fatal opioid overdose rate, and natural log of the baseline rate of the outcome. The natural log of the population size during the comparison period within a community is used as an offset. Adjusted rate is expressed per 100 000 residents.

^b^ Estimated dispersion parameter, k: 0.0074; 95% CI, 0.0031-0.0176.

^c^ Estimated dispersion parameter, k: 0.0182; 95% CI, 0.0083-0.0397.

^d^ Results obtained from a negative binomial model adjusting for intervention, state (Kentucky, Massachusetts, New York, Ohio), urban/rural classification, baseline fatal opioid overdose rate, and baseline rate of the outcome. The natural log of the population size during the comparison period within a community is used as an offset. Adjusted rate is expressed per 100 000 adult residents.

^e^ Estimated dispersion parameter, k: 0.0734; 95% CI, 0.0380-0.1419.

^f^ Estimated dispersion parameter, k: 0.0401; 95% CI, 0.0141-0.1140.

^g^ Results obtained from a negative binomial model adjusting for intervention, state (Kentucky, Massachusetts, New York, Ohio), urban/rural classification, baseline fatal opioid overdose rate, and baseline rate of the outcome. The natural log of the providers with a DATA 2000 waiver during the comparison period within a community is used as an offset. Adjusted rate is expressed per 100 providers with a DATA 2000 waiver.

^h^ Estimated dispersion parameter, k: 0.0026 (no 95% CI is given because multiple imputation was used).

### Practitioners With a DATA 2000 Waiver Who Actively Prescribed Buprenorphine

During the baseline period, the mean (SD) rate of practitioners who actively prescribed buprenorphine per 100 practitioners with a DATA 2000 waiver was 53.2 (16.1) among intervention and 54.7 (18.3) among wait-list control communities (eFigure in Supplement 3). Descriptive baseline statistics for rates are also provided by state and urban/rural classification (Table 1).

During the comparison period, the mean (SD) rate of practitioners who actively prescribed buprenorphine with a DATA 2000 waiver was 47.3 (15.1) among intervention and 47.7 (16.2) among wait-list control communities (eFigure in Supplement 3). Descriptive statistics for these rates are also provided by state, urban/rural classification, and patient-limit in Table 4.

**Table 4.** Number, Rate, and Relative Rate Between Intervention and Wait-List Control Communities of Practitioners With a DATA 2000 Waiver Who Actively Prescribe Buprenorphine

Group	Intervention				Wait-list control				Relative rate^a^
	Communities, No. (%)^b^	No. of practitioners with a DATA 2000 waiver actively prescribing buprenorphine, mean (SD)^c^	No. of practitioners with a DATA 2000 waiver, mean (SD)^d^	Rate, Mean (SD)^e^	Communities, No. (%)^b^	No. of practitioners with a DATA 2000 waiver actively prescribing buprenorphine, mean (SD)^c^	No. of practitioners with a DATA 2000 waiver, mean (SD)^d^	Rate, mean (SD)^e^	
Overall	33 (97.1)	58.1 (83.4)	126.5 (186.1)	47.3 (15.1)	30 (90.9)	55.3 (103.6)	118.3 (228.1)	47.7 (16.2)	0.99
State									
Kentucky	8 (100.0)	44.9 (65.1)	90.4 (135.1)	53.5 (8.8)	8 (100.0)	23.8 (51.9)	60.6 (139.2)	42.3 (18.6)	1.27
Massachusetts	8 (100.0)	30.9 (29.3)	69.3 (62.1)	42.6 (22.4)	7 (87.5)	56.1 (43.0)	116.4 (90.6)	51.2 (9.9)	0.83
New York	8 (100.0)	59.8 (66.9)	113.1 (123.8)	49.9 (17.2)	8 (100.0)	51.9 (55.9)	98.1 (111.3)	56.2 (9.3)	0.89
Ohio	9 (90.0)	92.7 (131.0)	221.6 (301.4)	43.5 (8.3)	7 (77.8)	94.6 (200.1)	209.1 (437.7)	41.0 (21.5)	1.06
Urban/rural classification									
Urban	18 (94.7)	94.0 (99.9)	205.9 (223.8)	48.7 (11.7)	18 (94.7)	80.9 (127.9)	175.3 (282.0)	50.8 (10.6)	0.96
Rural	15 (100.0)	15.1 (10.6)	31.3 (22.9)	45.6 (18.7)	12 (85.7)	17.0 (17.5)	32.8 (31.7)	43.2 (21.9)	1.05
Patient limit									
30	27 (79.4)	34.3 (52.4)	99.6 (141.5)	32.8 (15.1)	27 (81.8)	29.4 (51.6)	86.6 (161.1)	31.5 (16.0)	1.04
100	28 (82.4)	20.1 (25.1)	33.1 (41.9)	58.6 (20.1)	24 (72.7)	21.4 (34.3)	33.1 (50.5)	61.9 (19.8)	0.95
275	24 (70.6)	15.8 (15.9)	18.6 (19.6)	87.3 (10.4)	25 (75.8)	13.2 (25.2)	15.3 (30.1)	90.7 (20.6)	0.96

Abbreviations: DATA, Drug Addiction Treatment Act; FDA, Food and Drug Administration; OUD, opioid use disorder.

^a^ Relative rate in the specified group was calculated as the ratio of the mean rate of practitioners with a DATA 2000 waiver who actively prescribe buprenorphine products that are FDA-approved for OUD to state residents in the specified intervention group during the comparison period to the mean rate of practitioners with a DATA 2000 waiver who actively prescribe buprenorphine products that are FDA-approved for OUD to state residents in the specified wait-list control group during the comparison period.

^b^ Number of communities (% randomized) in the specified group that are included. Communities in the specified group that have suppressed event and/or population data are not included.

^c^ Mean (SD) of the number of practitioners with a DATA 2000 who actively prescribe buprenorphine products that are FDA-approved for OUD to state residents in the specified group during the comparison period.

^d^ Mean (SD) of the number of practitioners with a DATA 2000 waiver in the specified group during the comparison period.

^e^ Mean (SD) of the rate of practitioners with a DATA 2000 waiver who actively prescribe buprenorphine products that are FDA-approved for OUD to state residents in the specified group during the comparison period. Rate in the specified group within a community was calculated as the ratio of the number of practitioners with a DATA 2000 waiver who actively prescribe buprenorphine products that are FDA-approved for OUD to state residents in the specified group within a community to the number of practitioners with a DATA 2000 waiver in the specified group within a community multiplied by 100.

The absolute number of practitioners with a DATA 2000 waiver who actively prescribed buprenorphine with a DATA 2000 waiver and the raw rate per 100 practitioners with a DATA 2000 waiver among intervention and wait-list control communities are included in eTable 3 in Supplement 3. Descriptive statistics for rates are also provided by state, urban/rural classification, and patient-limit.

In adjusted analyses of the ITT population, there was no evidence of a significant difference in the rate of practitioners with a DATA 2000 waiver who actively prescribed buprenorphine between intervention and wait-list control communities (ARR, 1.00; 95% CI, 0.91-1.10) (Table 3). There was also no evidence to suggest that the effect of the CTH intervention compared with wait-list control on the rate of practitioners with a DATA 2000 waiver who actively prescribed buprenorphine differed significantly by state nor by urban/rural classification and patient limit (FDR-adjusted *P* = .88, *P* = .96 and *P* = .96 for interaction, respectively).

### DATA 2000 Waiver Trainings and Strategies to Increase Buprenorphine Prescribing

During the CTH intervention period, 64 buprenorphine DATA 2000 waiver trainings were conducted across all intervention communities. Massachusetts reported the most MOUD waiver trainings (49), followed by Kentucky (8), New York (5), and Ohio (2). Ten Beyond the Waiver trainings were documented, with 6 in Massachusetts and 4 in New York.

In addition, several strategies were designed to expand access to waivered practitioners, which included the placement of staff to expand existing programs, providing practitioners with technical assistance and ancillary support services, and encouraging use of free online waiver trainings. Other trainings were completed outside of the CTH framework.

## Discussion

In this randomized clinical trial, we assessed whether the CTH intervention was effective at increasing the rate of buprenorphine-waivered practitioners and active buprenorphine prescribing compared with treatment as usual. We found no significant differences in either outcome between the intervention and wait-list control communities. While the direction of the ARRs were in the hypothesized direction for some states, none were statistically significant. We found no evidence to suggest that the CTH intervention and related trainings increased waivered practitioner rates or active buprenorphine prescriber rates.

Our findings indicate that community-based strategies, including direct (eg, waiver trainings) and indirect (eg, technical assistance, ancillary support services, launching new programs), were insufficient to increase waivered practitioners and buprenorphine prescribing. While there were increases in the number of buprenorphine-waivered practitioners over the course of the study period, these increases in counts of waivered practitioners were evident in both intervention and wait-list control communities. In addition, as noted in another recent study focused on US clinicians, the increase in buprenorphine-waivered practitioners over recent years is contrasted with decreasing proportions of waivered practitioners actually prescribing buprenorphine.^38^ In fact, assessment of buprenorphine waivers and prescribing trends in the US between 2017 and 2021 found the number of waivered practitioners increased by 16% while the proportion prescribing buprenorphine decreased.^39^

Opioid use disorder is the only chronic medical illness for which access to highly effective medications has been restricted at the federal and state levels. Methadone can be dispensed only through opioid treatment programs. Until passage of the MAT Act, buprenorphine could be prescribed only by clinicians who completed a prerequisite training and were assigned a special DATA-Waiver registration number, in addition to the standard DEA number. Clinician level barriers to buprenorphine prescribing that hinder initiation of prescribing and contribute to relatively few patients treated by most prescribers need to be addressed. Barriers identified in earlier studies included the complexity of the waiver process itself, the lack of professional support and referral networks, challenges in getting started, and accessing reimbursement for treatment. Multilevel barriers to buprenorphine prescribing point to the need for multilevel interventions at the practitioner, health system, medical education, and policy levels to ensure low-barrier, widely accessible, and equitable access to this standard of care treatment.^40,41,42^ Such multilevel approaches need to motivate individual change, sharing success stories, humanizing patients with OUD, and implementing changes to medical education, health care delivery, and health policy changes.^40^ In the nonwaiver era, expanding and increasing support for practitioners from other parts of the health care system, such as behavioral health clinicians and pharmacists, and using a whole health model merits consideration.^43^

The impact of the COVID-19 pandemic may also help to explain our null findings. While in the US overall, the number of newly waivered clinicians slowed initially during the pandemic, the number of clinicians with 100- and 275-patient limit waivers increased.^41^ In our study, rates of waivered prescribers increased in both study conditions from baseline to the comparison period, but there were no significant differences between the 2 conditions during the comparison period. In addition, the weekly number of individuals filling buprenorphine prescriptions in the US plateaued relative to prepandemic rate increases.^44^ In our study, the proportion of waivered prescribers who prescribed buprenorphine decreased between the baseline and comparison periods; thus, increases in waivered prescribers did not increase prescription rates. These observed trends in the general US population and our results suggest that clinicians who were already prescribing made efforts to continue and expand, including through telemedicine,^45^ but that the unprecedented COVID-19–related burdens on the health care system may have slowed the pace of clinicians initiating buprenorphine prescribing.

Federal regulatory barriers to buprenorphine prescribing have been lowered recently,^18^ providing opportunities to enhance this activity. New interventions must be developed to encourage identification and engagement of patients with OUD, to motivate practitioners to prescribe, and to prioritize OUD treatment at all levels within the health care system. This will require a detailed and nuanced understanding of barriers to buprenorphine prescribing within the broader system, including state and federal agencies (eg, Centers for Medicare & Medicaid Services), payers, legislators, and health care systems that together shape clinical practice priorities and treatments offered by clinicians. Remaining barriers, many of which have existed for the past 2 decades,^46^ are certain to be complex; vary by practitioner, system, and geography; and involve issues as fundamental as stigma, culture, and lack of clinical support.^42^ While the CTH provides a multipronged, rigorous, and comprehensive implementation science approach, a more sophisticated tiered approach focused on increasing access to MOUD is especially needed at the present to accelerate development and testing of effective interventions at multiple levels. Changes to medical education, for instance, as recognized by federal leaders,^47^ have begun in some HCS states,^48^ and this is an important strategy for increasing motivation, providing newly trained clinicians with evidence-based training that may increase buprenorphine prescribing practices.^49^ In addition, expanding on the Veterans Health Administration implementation science approach and to prescribing MOUD merits attention, including its person-centered and medication first approaches, and efforts to expand MOUD options (eg, injectable buprenorphine), as well as their efforts to identify and address barriers and facilitators to MOUD among Veterans Health Administration veterans involved with the legal system.^50,51^ Some states have mandated that all forms of MOUD be available to individuals in carceral settings and that Medicaid be expanded to cover these individuals.^52^ Office-based addiction treatment programs, which have been successful, also should be considered.^53^

### Limitations

Our study has several limitations. Our findings may not be generalizable to all communities in the US. Second, our measure for active buprenorphine prescribing was limited to medication prescribed to 1 or more patients during the comparison period, which does not quantify prescriptions per prescriber. However, our stratified analyses assessed active buprenorphine prescribing among waivered practitioners with varying patient limits, allowing us to assess potential differences. Nevertheless, future analyses with more granular buprenorphine prescription levels are recommended, including buprenorphine administered at treatment facilities that are not reportable to PDMPs. Third, stratified analyses by race and ethnicity were not possible with the available data. Fourth, if a clinician moved from one HCS community to another during the comparison period, we counted them toward the clinician count in both communities. We acknowledged that they were active at some point in each community in providing access to buprenorphine treatment. While this was exceedingly rare, it may be a source of contamination that further tempered potential intervention effect in intervention vs wait-list control communities. Fifth, the CTH is a community-engaged, data-driven intervention, which resulted in variability in the implementation of practitioner training and training approaches across communities.

## Conclusions

In this randomized clinical trial, we found that the CTH intervention was not associated with increases in the rate of clinicians with a DATA 2000 waiver nor with rates of buprenorphine prescribing among waivered practitioners. A thorough understanding and mitigation of all barriers impacting buprenorphine expansion is urgently needed to encourage health care systems and clinicians to provide equitable and broad access to buprenorphine treatment.

## Notes

Supplement 1.
Trial Protocol

Supplement 2.
Statistical Analysis Plan

Supplement 3. **eFigure.** Raw Rate of DATA 2000 Waivered Providers and Waivered Providers Who Actively Prescribed Buprenorphine by Patient Level in Intervention and Wait-List Control Communities During Baseline and Comparison Periods **eTable 1.** HEALing Communities Study Sites and Underlying Population Sizes by State and Urban/Rural Classification in the Intention-to-Treat Population During the Comparison Period **eTable 2.** Overall Number, Rate, and Relative Rate Between Intervention and Wait-List Control Communities of Providers With a DATA 2000 Waiver in Intervention and Wait-List Control Communities by State, Urban/Rural Classification, and Patient Limit in the Intention-to-Treat Population During the Comparison Period **eTable 3.** Overall Number, Rate, and Relative Rate Between Intervention and Wait-List Control Communities of Providers With a DATA 2000 Who Actively Prescribe Buprenorphine for OUD to State Residents by State, Urban/Rural Classification, and Patient Limit in the Intention-to-Treat Population During the Comparison Period

Supplement 4.
Data Sharing Statement
