# Errors in Definitions Used by Research Sites

**DOI:** 10.1001/jamanetworkopen.2024.33598

**Published:** 2024-08-19

In the Original Investigation, “Impact of the Communities That HEAL Intervention on Buprenorphine-Waivered Practitioners and Buprenorphine Prescribing: A Prespecified Secondary Analysis of the HCS Randomized Clinical Trial,”^1^ that was published on February 22, 2024, in *JAMA Network Open*, the authors have reported errors requiring correction. After publication, the HCS Data Coordinating Center (DCC) for this study was informed on March 5, 2024, by research partners, about a potential issue with the source data sent from the 4 states in the analysis. Complete data were received by the 4 research sites and sent to the HCS DCC, and analyses were re-run by the HCS DCC in accordance with the original statistical analysis plan. This resulted in corrections to some counts, effect sizes, 95% CIs, and *P* values. The corrected findings did not change the direction of associations, statistical significance, or interpretations. The authors have explained the errors and corrections in a Comment posted with the corrected article.^2^
