# Supplementary material for: Impact of the Communities That HEAL Intervention on Buprenorphine-Waivered Practitioners and Buprenorphine Prescribing: A Prespecified Secondary Analysis of the HCS Randomized Clinical Trial
Source: JAMA Netw Open. 2024 Feb 22;7(2):e240132. doi: 10.1001/jamanetworkopen.2024.0132 (PMC10884876; doi:10.1001/jamanetworkopen.2024.0132)
Supplement: Supplement 2. — Statistical Analysis Plan [file jamanetwopen-e240132-s002.pdf]

STATISTICAL ANALYSIS PLAN

Do communities implementing the Communities That Heal (CTH) intervention have significantly more providers with Drug Addiction Treatment Act of 2000 (DATA 2000) waivers?

Proposal Number: MP0055

SAP VERSION: Version 3.0

SAP DATE: July 19, 2023

PI/SPONSOR: Sharon Walsh, PhD (University of Kentucky)
Jeffrey Samet, MD (Boston University)
Nabila El-Bassel, PhD (Columbia University)
Bridget Freisthler (Ohio State University)
Emmanuel Oga, MD (RTI International)
Gary Zarkin, PhD (RTI International)
LaShawn Glasgow, PhD (RTI International)
Redonna Chandler, PhD (NIDA)
Jennifer Villani, PhD (NIDA)

PREPARED BY: RTI International and the HCS Statistics and Data Analysis Work Group (StaDA-WG).

AUTHORS: Denise Babineau, Lauren D'Costa, and Rouba Chahine for the HEALing Communities Study Data Coordinating Center Statistical Operations (DCC StatOps) group.

SAP REVIEWERS: Philip Westgate (University of Kentucky), Debbie Cheng (Boston University), Daniel Feaster (University of Miami), Soledad Fernandez (Ohio State University) for the HCS Statistics and Data Analysis Work Group (StaDA-WG). Raymond R. Balise (University of Miami) for the SAP Writing Team.

Contents

1 ADMINISTRATIVE INFORMATION 4
1.1 SAP REVISION HISTORY 4
1.2 LIST OF ABBREVIATIONS 5
2 INTRODUCTION 5
2.1 OBJECTIVES 6
2.1.1 Objective 1 6
2.1.2 Objective 2 6
2.1.3 Objective 3 6
2.1.4 Objective 4 6
2.1.5 Objective 5 6
2.2 HYPOTHESES 6
2.2.1 Hypothesis 1 6
2.2.2 Hypothesis 2 6
2.2.3 Hypothesis 3 7
2.2.4 Hypothesis 4 7
2.2.5 Hypothesis 5 7
3 STUDY METHODS 8
3.1 TRIAL DESIGN 8
3.2 RANDOMIZATION 8
3.3 STATISTICAL INTERIM ANALYSIS AND STOPPING GUIDANCE 8
3.4 TIMING OF ANALYSIS 8
4 STATISTICAL PRINCIPLES 8
4.1 CONFIDENCE INTERVALS AND P-VALUES 8
4.2 ANALYSIS POPULATIONS 9
5 STATISTICAL ANALYSIS 9
5.1 OUTCOME DEFINITIONS 9
5.2 ANALYSIS METHODS 10
5.2.1 Statistical Methods 10
5.2.2 Assumption Checks 12
5.2.3 Alternate Analysis Methods 12

5.2.4 Sensitivity Analysis *12*

5.2.5 Subgroup Analysis *12*

5.3 MISSING DATA 14

5.4 ADDITIONAL ANALYSES 14

5.5 STATISTICAL SOFTWARE 15

5.6 LIST OF POTENTIAL DISPLAYS 15

6 REFERENCES 17

7 APPENDICES 18

7.1 APPENDIX 1: STUDY RANDOMIZATION 18

1 ADMINISTRATIVE INFORMATION

1.1 SAP Revision History

Ver.	Justification for change	Date
1.0	Initial Version	2023-01-13
2.0	• Revised PI/Sponsor list on title page<br>• Provided a more detailed description of the modeling approach that will be used for testing the effect of the CTH intervention on each outcome<br>• Clarified the time period during which the baseline opioid overdose death rate, the baseline rate of the outcome, and the offset term will be measured<br>• Clarified reporting of subgroup analyses<br>• Provided a more detailed description of the modeling approach that will be used to address missing data<br>• Revised list of potential displays	2023-04-19
3.0	• Modified Section 5.6 to indicate that descriptive statistics of baseline characteristics in Table 1 will be provided for levels of the stratification variable of interest.<br>• Modified Section 5.2.5 to indicate that reporting of subgroup analyses will include adjusted rates and relative rates between Wave 1 and 2 within each level of the stratification variable, regardless of the statistical significance of the interaction test.	2023-07-19

1.2 List of Abbreviations

Abbreviation:	Definition:
$	Dollar
%	Percent
±	Plus or Minus
≤	Less than or equal to
<	Less than
=	Equals
≥	Greater than or equal to
>	Greater than
CI	Confidence Interval
CTH	Communities That HEAL
DATA 2000	Drug Addiction Treatment Act of 2000
DCC	Data Coordinating Center
DCC StatOps	Data Coordinating Center Statistical Operations
DEA	Drug Enforcement Administration
EBP	Evidence-Based Program
FDR	False Discovery Rate
HCS	HEALing Communities Study
ITT	Intention-To-Treat
KY	Kentucky
MA	Massachusetts
NIDA	National Institute on Drug Abuse
NY	New York
OH	Ohio
ORCCA	Opioid-overdose Reduction Continuum of Care Approach
OUD	Opioid use disorder
PDMP	Prescription Drug Monitoring Program
PP	Per-Protocol
RS	Research site
SAP	Statistical Analysis Plan

2 INTRODUCTION

The HEALing Communities Study (HCS)[1] is a 5-year, multi-site, parallel group, cluster randomized wait-list comparison trial to test the impact of implementing an integrated set of evidence-based practices from the Communities That HEAL (CTH) framework delivered across healthcare, behavioral health, justice, and other community-based settings in highly affected communities. The goal of the HCS is to reduce opioid overdose deaths.

This document outlines the statistical analysis plan (SAP) that will be followed to address the research objectives that are of interest in MP0055.

2.1 Objectives

2.1.1 Objective 1

To compare the rate of providers with a Drug Addiction Treatment Act of 2000 (DATA 2000) waiver per 100,000 adults during the evaluation period between Waves 1 and 2.

2.1.2 Objective 2

To compare the rate of providers with a DATA 2000 waiver with a 30-patient limit per 100,000 adults during the evaluation period between Waves 1 and 2.

2.1.3 Objective 3

To compare the rate of providers with a DATA 2000 waiver with a 100-patient limit per 100,000 adults during the evaluation period between Waves 1 and 2.

2.1.4 Objective 4

To compare the rate of providers with a DATA 2000 waiver with a 275-patient limit per 100,000 adults during the evaluation period between Waves 1 and 2.

2.1.5 Objective 5

To compare the rate of active buprenorphine prescribing per 100 providers with a DATA 2000 waiver during the evaluation period between Wave 1 and Wave 2.

2.2 Hypotheses

2.2.1 Hypothesis 1

We expect that there will be a difference in the rate of providers with a DATA 2000 waiver per 100,000 adults during the evaluation period between Wave 1 and Wave 2. In particular, we expect that the rate of providers with a DATA 2000 waiver per 100,000 adults during the evaluation period in Wave 1 is <u>higher</u> than Wave 2.

The null (H_0) and alternative (H_A) statistical hypotheses are:

> H_0: The rate of providers with a DATA 2000 waiver per 100,000 adults during the evaluation period from July 1, 2021 to June 30, 2022 in Wave 1 is equal to Wave 2.
>
> H_A: The rate of providers with a DATA 2000 waiver per 100,000 adults during the evaluation period from July 1, 2021 to June 30, 2022 in Wave 1 is not equal to Wave 2.

2.2.2 Hypothesis 2

We expect that there will be a difference in the rate of providers with a DATA 2000 waiver with a 30-patient limit per 100,000 adults during the evaluation period between Wave 1 and Wave 2. In

particular, we expect that the rate of providers with a DATA 2000 waiver with a 30-patient limit per 100,000 adults during the evaluation period in Wave 1 is higher than Wave 2.

The statistical hypotheses are:

H_0: The rate of providers with a DATA 2000 waiver with a 30-patient limit per 100,000 adults during the evaluation period from July 1, 2021 to June 30, 2022 in Wave 1 is equal to Wave 2.

H_A: The rate of providers with a DATA 2000 waiver with a 30-patient limit per 100,000 adults during the evaluation period from July 1, 2021 to June 30, 2022 in Wave 1 is not equal to Wave 2.

2.2.3 Hypothesis 3

We expect that there will be a difference in the rate of providers with a DATA 2000 waiver with a 100-patient limit per 100,000 adults during the evaluation period between Wave 1 and Wave 2. In particular, we expect that the rate of providers with a DATA 2000 waiver with a 100-patient limit per 100,000 adults during the evaluation period in Wave 1 is higher than Wave 2.

The statistical hypotheses are:

H_0: The rate of providers with a DATA 2000 waiver with a 100-patient limit per 100,000 adults during the evaluation period from July 1, 2021 to June 30, 2022 in Wave 1 is equal to Wave 2.

H_A: The rate of providers with a DATA 2000 waiver with a 100-patient limit per 100,000 adults during the evaluation period from July 1, 2021 to June 30, 2022 in Wave 1 is not equal to Wave 2.

2.2.4 Hypothesis 4

We expect that there will be a difference in the rate of providers with a DATA 2000 waiver with a 275-patient limit per 100,000 adults during the evaluation period between Wave 1 and Wave 2. In particular, we expect that the rate of providers with a DATA 2000 waiver with a 275-patient limit per 100,000 adults during the evaluation period in Wave 1 is higher than Wave 2.

The statistical hypotheses are:

H_0: The rate of providers with a DATA 2000 waiver with a 275-patient limit per 100,000 adults during the evaluation period from July 1, 2021 to June 30, 2022 in Wave 1 is equal to Wave 2.

H_A: The rate of providers with a DATA 2000 waiver with a 275-patient limit per 100,000 adults during the evaluation period from July 1, 2021 to June 30, 2022 in Wave 1 is not equal to Wave 2.

2.2.5 Hypothesis 5

We expect that there will be a difference in the rate of actively prescribing buprenorphine per 100 providers with a DATA 2000 waiver during the evaluation period between Wave 1 and Wave 2. In particular, we expect that the rate of actively prescribing buprenorphine per 100 providers with a DATA 2000 waiver during the evaluation period is higher for Wave1 than Wave 2. For the purposes of this analysis, active prescribing is defined as ≥1 prescription(s) during the evaluation period.

The statistical hypotheses are:

H_0: The rate of actively prescribing buprenorphine per 100 providers with a DATA 2000 waiver during the evaluation period from July 1, 2021 to June 30, 2022 in Wave 1 is equal to Wave 2.

H_A: The rate of actively prescribing buprenorphine per 100 providers with a DATA 2000 waiver during the evaluation period from July 1, 2021 to June 30, 2022 in Wave 1 is not equal to Wave 2.

3 STUDY METHODS

This section contains information about the study design and statistical analysis that will be performed to assess the research objectives and statistical hypotheses outlined in Section 2.

3.1 Trial design

The HCS is a multi-site, parallel arm, cluster randomized wait-list comparison trial composed of 67 communities from four Research Sites (RS): Kentucky (KY), Massachusetts (MA), New York (NY), and Ohio (OH). Communities were randomly assigned to the CTH intervention arm (Wave 1, N = 34 communities) or the wait-list comparison arm (Wave 2, N = 33 communities).

3.2 Randomization

The 67 HCS communities were randomly assigned to Wave 1 (CTH intervention) or Wave 2 (wait-list control) according to Appendix 1. Randomization was stratified by RS. For each RS, covariate-constrained randomization (Moulton, 2004)[2] was used to ensure balance between Wave 1 and Wave 2 communities on three key community characteristics at baseline:

1. opioid overdose death rate averaged over 2016 and 2017
2. population size
3. urban versus rural status.

Given the nature of the research, no blinding was performed in this study.

3.3 Statistical Interim Analysis and Stopping Guidance

There are no interim efficacy analyses, safety analyses, or interim stopping rules that must be considered for this SAP.

3.4 Timing of Analysis

The evaluation period for the CTH intervention is from July 1, 2021 through June 30, 2022. Data collection for the evaluation period is expected to be complete within 6-9 months from the end of the evaluation period. Subsequent data cleaning and analysis will be completed within 3-6 months and dissemination of results will take place immediately after.

4 STATISTICAL PRINCIPLES

4.1 Confidence Intervals and P-values

All statistical computations will be performed by HCS biostatisticians from the Data Coordinating Center Statistical Operations group (DCC StatOps). For descriptive summaries of study data, the following will be presented:

- Nominal/categorical measures will be summarized using frequencies and percentages;
- Interval or ratio scale measures will be summarized using means, standard deviations, medians, 95% confidence intervals (CIs), 25th and 75th percentiles, and ranges;

- Ordinal measures will be summarized depending on the number of levels. An ordinal measure with five levels or less will be summarized as a nominal measure. An ordinal measure with more than five levels will be summarized as an interval or ratio scale measure.

The balance or imbalance of these characteristics will be studied and reported, particularly for analyses comparing the two trial arms. Graphical displays will be used to show distributions (box plots, density curves). The reported p-values will be based on two-sided tests at an $\alpha=0.05$ unless otherwise specified. When p-value correction is appropriate, Benjamini-Hochberg (1995)[3] False Discovery Rate (FDR) adjustments will be used to address multiplicity and preserve Type I error rate.

Unless required otherwise by a journal, the following rules are standard:

- Moment statistics including mean and standard deviation will be reported at 1 more significant digit than the precision of the data.
- Order statistics including median, minimum, and maximum values will be reported to the same level of precision as the original observations. If any values are calculated to have more significant digits, then the value should be rounded so that it is the same level of precision as the original data.
- Following SAS rules, the median will be reported as the average of the two middle numbers (by order) if the dataset contains an even number of observations.
- Test statistics including t and z test statistics will be reported to two decimal places.
- P-values will be reported to 3 decimal places if > 0.001. If less than 0.001, p-values will be reported as '<0.001'. P-values will be reported as 0.05 rather than .05.
- No preliminary rounding should be performed; rounding should only occur after analysis. To round, consider digit to right of last significant digit: if < 5 round down, if ≥ 5 round up.

4.2 Analysis Populations

The analyses of each outcome will be performed using an Intention-To-Treat (ITT) framework, defined as the inclusion of all communities randomized into the HCS and analyzed according to the trial arm to which the community was randomized.

If specified, a sensitivity analysis of an outcome will also be performed using a Per-Protocol (PP) framework, defined as the inclusion of all randomized communities who complete the HCS protocol with no major deviations and analyzed according to the trial arm to which the community was randomized.

For both ITT and PP analysis populations, the outcomes that will be used to address Objectives 1-4 will be measured using the number of individuals within a community while the outcome that will be used to address Objective 5 will be measured using the number of providers within a community.

5 STATISTICAL ANALYSIS

5.1 Outcome Definitions

Table 1 provides the definitions for the outcomes that will be used to address the objectives listed in Section 2.1.

Table 1. Outcome Definitions

Objective	Outcome	Technical Specifications Ref.	Source
1	Number of providers with a DATA 2000 waiver	3.2	DEA
2	Number of providers with a DATA 2000 waiver with a 30-patient limit	3.2.30	DEA
3	Number of providers with a DATA 2000 waiver with a 100-patient limit	3.2.100	DEA
4	Number of providers with a DATA 2000 waiver with a 275-patient limit	3.2.275	DEA
5	Number of providers with a DATA 2000 waiver who actively prescribe buprenorphine products that are FDA approved for opioid use disorder (OUD) to state residents	3.3	PDMP/ DEA

5.2 Analysis Methods

5.2.1 Statistical Methods

The models used to address the objectives in Section 2.1 will test the effect of the CTH intervention on each outcome between Wave 1 and Wave 2 communities using methods described in Westgate et al (2022)[4]. To address each objective, a negative binomial or Poisson regression model will be fit to the corresponding outcome in Section 5.1 as the dependent variable with trial arm (Wave 1 versus Wave 2) included as the main independent variable. Table 2 provides a description of the dependent variable that will be used to address each objective, the statistical model that will be fit, the offset term that will be included in the model, and a list of covariates that will be included as fixed effects in the model. Small sample adjusted empirical standard error estimates will be applied using the average of the small-sample corrected empirical estimators proposed by Mancl and DeRouen (2001)[5] and Kauermann and Carroll (2001)[6] (SAS options given by “empirical=FIRORES” and “empirical=root”, respectively) and degrees of freedom equal to the number of communities minus the number of regression parameters. In the unlikely event this estimator is computationally infeasible, one of the following SAS options will be used: “empirical=FIRORES”, “empirical=FIROEEQ”, or “empirical=DF”. If needed, a similar test can be conducted for each of the individual parameters described above.

Table 2. Statistical Models

Objective	Dependent Variable	Model	Offset	Covariates
1	Number of providers with a DATA 2000 waiver during the evaluation period within a community	Negative binomial (or Poisson if indicated during model fit)	Natural log of the community population size of individuals 18 years of age or older during the evaluation period[1]	1. RS (KY, MA, NY, OH)<br>2. Geographic location (rural or urban)<br>3. Natural log of the baseline opioid overdose death rate per 100,000 adults[2,6]<br>4. Natural log of the baseline rate of providers with a DATA 2000 waiver per 100,000 adults[3,6]
2	Number of providers with a DATA 2000 waiver with a 30-patient limit during the evaluation period within a community	Negative binomial (or Poisson if indicated during model fit)	Natural log of the community population size of individuals 18 years of age or older	1. RS (KY, MA, NY, OH)<br>2. Geographic location (rural or urban)

Objective	Dependent Variable	Model	Offset	Covariates
			during the evaluation period[1]	3. Natural log of the baseline opioid overdose death rate per 100,000 adults[2,6]<br>4. Natural log of the baseline rate of providers with a DATA 2000 waiver with a 30-patient limit per 100,000 adults[3,6]
3	Number of providers with a DATA 2000 waiver with a 100-patient limit during the evaluation period within a community	Negative binomial (or Poisson if indicated during model fit)	Natural log of the community population size of individuals 18 years of age or older during the evaluation period[1]	1. RS (KY, MA, NY, OH)<br>2. Geographic location (rural or urban)<br>3. Natural log of the baseline opioid overdose death rate per 100,000 adults[2,6]<br>4. Natural log of the baseline rate of providers with a DATA 2000 waiver with a 100-patient limit per 100,000 adults[3,6]
4	Number of providers with a DATA 2000 waiver with a 275-patient limit during the evaluation period within a community	Negative binomial (or Poisson if indicated during model fit)	Natural log of the community population size of individuals 18 years of age or older during the evaluation period[1]	1. RS (KY, MA, NY, OH)<br>2. Geographic location (rural or urban)<br>3. Natural log of the baseline opioid overdose death rate per 100,000 adults[2]<br>4. Natural log of the baseline rate of providers with a DATA 2000 waiver with a 275-patient limit per 100,000 adults[3,6]
5	Number of providers with a DATA 2000 waiver who actively prescribe buprenorphine products that are FDA approved for OUD to state residents during the evaluation period within a community	Negative binomial (or Poisson if indicated during model fit)	Natural log of the number of providers with a DATA 2000 waiver during the evaluation period within a community[4]	1. RS (KY, MA, NY, OH)<br>2. Geographic location (rural or urban)<br>3. Natural log of the baseline opioid overdose death rate per 100,000 adults[2]<br>4. Natural log of the baseline rate of actively prescribing buprenorphine per 100 providers with a DATA 2000 waiver[5,6]

1. The population that will be used for the offset:
    - P.1.1: 2020 county-defined community population denominator, 18+
    - P.2.1: 2021 zip code-defined community population denominator, 18+
2. The baseline opioid overdose death rate per 100,000 adults will be calculated using the ratio of the number of opioid overdose deaths as measured from January 2019 to December 2019 within a community to the community population size of individuals 18 years of age or older measured during 2020 (county-defined) or 2021 (zip-code defined) multiplied by 100,000. If there are any communities where the baseline opioid overdose death rate is 0, the log transformation cannot be applied and so the baseline opioid overdose death rate will be used instead.
3. The baseline rate of providers with a DATA 2000 waiver for the given patient limit (overall or 30, 100, or 275) per 100,000 adults will be calculated using the ratio of the number of providers with a DATA 2000 waiver for the given patient limit measured from January 2019 to December 2019 within a community to the community population size of individuals 18 years or older measured during 2020 (county-defined) or 2021 (zip-code defined) multiplied by 100,000. If there are any communities where the baseline rate of providers with a DATA 2000 waiver for the given patient limit (overall or 30, 100, or 275) is 0, the log transformation cannot be applied and so the baseline rate of providers with a DATA 2000 waiver for the given patient limit will be used instead.
4. Communities where there are no providers with a DATA 2000 waiver during the evaluation period will not be included in this analysis because a log transformation cannot be applied.
5. The baseline rate of actively prescribing buprenorphine per 100 providers with a DATA 2000 waiver will be calculated using the ratio of the number of providers with a DATA 2000 waiver who actively prescribe buprenorphine products that are FDA approved for OUD to state residents measured from January 2019 to December 2019 within a community to the number of providers with a DATA 2000 waiver measured from January 2019 to December 2019 within a community multiplied by 100. If there are any communities where the baseline rate of actively prescribing buprenorphine per 100 providers with a DATA 2000 waiver is 0, the log transformation cannot be applied and so the baseline rate of actively prescribing buprenorphine per 100 providers with a DATA 2000 waiver will be used instead. However, if there are any communities that have no providers with a DATA 2000 waiver during 2019, this rate cannot be estimated and so the rate will be removed as a covariate in the model.

6. In cases where a log transformation of the baseline rate of opioid overdose death rate (or baseline rate of the outcome) can be used as a covariate for the primary analysis but the untransformed baseline rate of opioid overdose death rate (or baseline rate of the outcome) must be used as a covariate for at least one subgroup analysis, use the untransformed baseline rate of opioid overdose death rate (or baseline rate of the outcome) to maintain consistency across models of the same outcome.

The results of the model for each outcome will be used to estimate and report:

- The adjusted relative rate (and 95% CI and p-value) of the outcome in the population of interest during the evaluation period from July 1, 2021 to June 30, 2022 between Wave 1 and Wave 2.
- The adjusted rate (and 95% CI) of the outcome in the population of interest during the evaluation period from July 1, 2021 to June 30, 2022 within Wave 1.
- The adjusted rate (and 95% CI) of the outcome in the population of interest during the evaluation period from July 1, 2021 to June 30, 2022 within Wave 2.

Note that adjusted rates and 95% CIs will be calculated using least squares means.

5.2.2 Assumption Checks

It is possible that for some outcomes, a Poisson model could be used in lieu of a negative binomial model. To evaluate this, the overdispersion parameter, k, of the negative binomial model will first be estimated using PROC GLIMMIX and its 95% CI will be estimated by refitting the model using PROC GENMOD as PROC GLIMMIX does not generate a CI for k. If the negative binomial is appropriate for the data collected, the value of k should reflect overdispersion and be a positive value, but if k is close to zero then GLIMMIX may fail to converge. If PROC GLIMMIX fails to converge, then k will be set to 0 and a working Poisson model will be fit to the data in PROC GLIMMIX.

Other model assumptions such as linearity will be assessed by plotting residuals from the models against the value of the linear predictor (predicted outcome values), and residuals versus values of covariates employed in the models (e.g., baseline opioid-related death rate). No obvious trends should be evident (i.e., uniform horizontal band present) and scatter of residuals should not depend on the linear predictor.

5.2.3 Alternate Analysis Methods

A negative binomial response is assumed (overdispersion parameter, $k > 0$). If this assumption does not hold, then a Poisson response may be adequate. If the negative binomial model does not fit adequately, as mentioned in Section 5.2.2, then k will be set to 0 and a Poisson model will be fit to the data.

5.2.4 Sensitivity Analysis

Each model specified in Section 5.2.1 will be replicated using a PP framework (as defined in Section 4.2).

5.2.5 Subgroup Analysis

There are 2 planned subgroup analyses for Objectives 1-4 using the following stratification variables:

- RS (KY, MA, NY, OH)
- Geographic location (rural or urban)

There are 3 planned subgroup analyses for Objective 5 using the following stratification variables:

- RS (KY, MA, NY, OH)
- Geographic location (rural or urban)
- DATA 2000 waiver patient limit (30 patients, 100 patients, 275 patients)

For each subgroup analysis, a separate model will be fit using an ITT framework. Each model will be similar to that used for the primary analysis described in Section 5.2.1 but will also include fixed effects for the corresponding stratification variable as well as an interaction between intervention and the stratification variable. The subgroup analysis using DATA 2000 waiver patient limits for Objective 5 will need to account for repeated measures across the 3 levels within a community and so a GEE-type negative binomial (or Poisson) regression model will be used.

To account for multiple comparisons arising from subgroup analyses that are performed, the FDR will be controlled across the entire set of tests (i.e., interaction test between intervention and the stratification variable as well as pairwise tests between levels of a stratification variable across all outcomes) using Benjamini-Hochberg (1995)[3] FDR-adjusted p-values.

Note that subgroup analyses for the community level variables, RS and geographic location, will use the same data structure as that used for the primary analysis. The subgroup analysis using DATA 2000 waiver patient limits for Objective 5 will require the data structure to be modified such that there is a separate record for each DATA 2000 waiver patient limit within a community. While design variables such as RS, geographic location, and baseline opioid overdose death rate will not change for each of these records, the dependent variable, offset, and baseline rate of the outcome of interest will need to be modified so that they are specific to each DATA 2000 waiver patient limit. Table 3 provides a detailed description for the subgroup analysis using DATA 2000 waiver patient limits for Objective 5.

Table 3. Data Structure for Subgroup Analysis using DATA 2000 waiver patient limit for Objective 5

Objective	Dependent Variable	Model	Offset	Covariates
5	Number of providers with a DATA 2000 waiver who actively prescribe buprenorphine products that are FDA approved for OUD to state residents during the evaluation period within a community, stratified by DATA 2000 waiver patient limit (30 patients, 100 patients, 275 patients)	Negative binomial (or Poisson if indicated during model fit)	Natural log of the number of providers with a DATA 2000 waiver during the evaluation period within a community, stratified by DATA 2000 waiver patient limit (30 patients, 100 patients, 275 patients)[1]	1. RS (KY, MA, NY, OH)<br>2. Geographic location (rural or urban)<br>3. Natural log of the baseline opioid overdose death rate per 100,000 adults[2,4]<br>4. Natural log of the baseline rate of actively prescribing buprenorphine per 100 providers with a DATA 2000 waiver per 100 providers, stratified by DATA 2000 waiver patient limit (30 patients, 100 patients, 275 patients)[3,4]

1. A log transformation cannot be applied to a 0 value. Consequently: 1) Any level of a stratification variable defined by a community where there are no providers with a DATA 2000 waiver with a 30-patient limit during the evaluation period will not be included in this analysis; 2) Any level of a stratification variable defined by a community where there are no providers with a DATA 2000 waiver with a 100-patient limit during the evaluation period will not be included in this analysis; and 3) Any level of a stratification variable defined by a community where there are no providers with a DATA 2000 waiver with a 275-patient limit during the evaluation period will not be included in this analysis.
2. The baseline overdose death rate per 100,000 adults will be calculated using the ratio of the number of opioid overdose deaths as measured from January 2019 to December 2019 within a community to the community population size of individuals 18 years of age or older measured during 2020 (county-defined) or 2021 (zip-code defined) multiplied by 100,000. If there are any communities where the baseline opioid overdose death rate is 0, the log transformation cannot be applied and so the baseline opioid overdose death rate will be used instead.

3. The baseline rate of actively prescribing buprenorphine per 100 providers with a DATA 2000 waiver will be calculated using the ratio of the number of providers with a DATA 2000 waiver who actively prescribe buprenorphine products that are FDA approved for OUD to state residents measured from January 2019 to December 2019 within a community to the number of providers with a DATA 2000 waiver measured from January 2019 to December 2019 within a community multiplied by 100. If there are any communities where the baseline rate of actively prescribing buprenorphine per 100 providers with a DATA 2000 waiver with a 30, 100, or 275 patient limit is 0, the log transformation cannot be applied and so the baseline rate of actively prescribing buprenorphine per 100 providers with a DATA 2000 waiver with a 30, 100, or 275 patient limit will be used instead. However, if there are any communities that have no providers with a DATA 2000 waiver with a 30, 100, or 275 patient limit during 2019, this rate cannot be estimated and so the rate will be removed as a covariate in the model.
4. In cases where a log transformation of the baseline rate of opioid overdose death rate (or baseline rate of the outcome) can be used as a covariate for the primary analysis but the untransformed baseline rate of opioid overdose death rate (or baseline rate of the outcome) must be used as a covariate for at least one subgroup analysis, use the untransformed baseline rate of opioid overdose death rate (or baseline rate of the outcome) to maintain consistency across models of the same outcome.

For each test of effect modification, adjusted rates and relative rates between Wave 1 and 2 within each level of a stratification variable will be reported in addition to the FDR-adjusted p-value associated with the interaction test. If the test for effect modification is not statistically significant at the 0.05 level, no further reporting will be performed. If the test for effect modification is statistically significant at the 0.05 level, the following will be reported:

- estimate and 95% CI of the ratio of the relative rate between any 2 levels of a stratification variable; and
- pairwise FDR-adjusted p-value between any 2 levels of a stratification variable.

5.3 Missing Data

Missing data may arise when outcome data is suppressed at an RS. For suppressed data at the community-level, multiple imputation for missing data (Rubin 1987[7]) will be performed using SAS PROC MI (SAS/STAT® 15.2 User's Guide[8]). Twenty imputations will be used for analysis (Allison 2012[9]). SAS PROC MIANALYZE (SAS/STAT® 15.2 User's Guide[8]) will be used to combine results across imputations Multiple imputation will not be performed to account for suppressed data at the level of a stratification variable within a community (e.g., RS, geographic location, or DATA 2000 waiver patient limit). In this case, levels of a stratification variable with suppressed data will be excluded from the analysis. Regardless of the approach taken, the amount of suppressed data will be summarized.

5.4 Additional Analyses

No additional analyses are planned, other than those pertaining to the Opioid-overdose Reduction Continuum of Care Approach (ORCCA). The ORCCA consists of three menus of evidence-based practices (EBPs) that were implemented in Wave 1 communities. Within each menu, EBPs were implemented in different combinations of strategies (e.g., active opioid overdose education and naloxone distribution at high-risk venues), sectors (e.g., healthcare), and venues (e.g., healthcare-emergency department). Each strategy-sector-venue combination is referred to as a triad. A triad is the primary identifier for a strategy that is planned and then implemented within a community. For each triad that is selected by a community, respondents were asked specific questions relating to:

- intent to reach special populations;
- development of an implementation plan for the strategy;
- initiation of the implementation plan;
- the number of partner organizations/practices that are implementing the strategy; and
- a brief description of the strategy.

To summarize this information across Wave 1 communities, Table 6 (see Section 5.6) may provide frequencies and percentages of strategy and sector for each of Menu 1: Overdose Education and Naloxone Distribution (OEND), Menu 2: Medication for Opioid Use Disorder (MOUD), and Menu 3: Safer Prescribing. Summaries will be presented by RS (KY, MA, NY, OH), by geographic location (urban, rural), and overall. The table will only include summary statistics on menus that are relevant to the manuscript. For this manuscript, only Menu 2 will be summarized.

Depending on the scope of the manuscript, frequencies and percentages for specific strategy-sector-venue strategies may also be included. Inclusion will be informed by: 1) the ORCCA Overview, which maps outcome measures to ORCCA menus and strategies; and 2) the sector and venue definitions provided in Appendix D of the ORCCAT SOP, which were vetted by the Common Implementation Metrics workgroup.

5.5 Statistical Software

The SAS statistical package (version 9.4 or higher) will be utilized for all analyses. The R software package may be used to create figures and other graphical displays.

5.6 List of Potential Displays

Listing of table shells and figures for specified analysis.

- Table 1. Baseline Demographic Characteristics of N=67 Communities Participating in the HEALing Communities Study by Wave (January 2019 – December 2019)
  - This table will be used to provide baseline demographic descriptive statistics in the ITT population.
  - Summary statistics will also be provided for each level of a stratification variable of interest.
- Table 2 series: This series of tables will be used to provide the means and SDs of the number of events and population for each outcome during the evaluation period by wave in the ITT population. Summary statistics will also be provided for each level of a stratification variable of interest.
  - Table 2a. Descriptive Means of Providers with a DATA 2000 Waiver During the Evaluation Period using the Intention-to-Treat Population
  - Table 2b. Descriptive Means of Providers with a DATA 2000 Waiver with a 30-Patient Limit During the Evaluation Period using the Intention-to-Treat Population
  - Table 2c. Descriptive Means of Providers with a DATA 2000 Waiver with a 100-Patient Limit During the Evaluation Period using the Intention-to-Treat Population
  - Table 2d. Descriptive Means of Providers with a DATA 2000 Waiver with a 275-Patient Limit During the Evaluation Period using the Intention-to-Treat Population
  - Table 2e. Descriptive Means of Providers with a DATA 2000 Waiver who Actively Prescribe Buprenorphine Products that are FDA approved for OUD to State Residents During the Evaluation Period using the Intention-to-Treat Population
- Table 2x. Descriptive Means of Efficacy Outcomes During the Evaluation Period using the Per-Protocol Population
  - This table will be used to provide the means and SDs of the number of events and population for each outcome during the evaluation period by wave in the PP

population. Summary statistics will not be provided for each level of a stratification variable of interest.

- Table 3 series: This series of tables will be used to provide the raw sum of the number of events and population for each outcome during the evaluation period by wave in the ITT population. Summary statistics will also be provided for each level of a stratification variable of interest.
    - Table 3a. Descriptive Sums of Providers with a DATA 2000 Waiver During the Evaluation Period using the Intention-to-Treat Population
    - Table 3b. Descriptive Sums of Providers with a DATA 2000 Waiver with a 30-Patient Limit During the Evaluation Period using the Intention-to-Treat Population
    - Table 3c. Descriptive Sums of Providers with a DATA 2000 Waiver with a 100-Patient Limit During the Evaluation Period using the Intention-to-Treat Population
    - Table 3d. Descriptive Sums of Providers with a DATA 2000 Waiver with a 275-Patient Limit During the Evaluation Period using the Intention-to-Treat Population
    - Table 3e. Descriptive Sums of Providers with a DATA 2000 Waiver who Actively Prescribe Buprenorphine Products that are FDA approved for OUD to State Residents During the Evaluation Period using the Intention-to-Treat Population
- Table 3x. Descriptive Sums of Efficacy Outcomes During the Evaluation Period using the Per-Protocol Population
    - This table will be used to provide the raw sum of the number of events and population for each outcome during the evaluation period by wave in the PP population. Summary statistics will not be provided for each level of a stratification variable of interest.
- Table 4a. Adjusted Rate of Each Outcome Within Waves and Adjusted Relative Rate of Each Outcome Between Wave 1 and Wave 2 Communities in the Intention-to-Treat Population
    - This table will include the results of the hypothesis tests corresponding to the CTH intervention effect on the outcomes in the ITT population.
- Table 4b. Adjusted Rate of Each Outcome Within Waves and Adjusted Relative Rate of Each Outcome Between Wave 1 and Wave 2 Communities in the Per-Protocol Population
    - This table will include the results of the hypothesis tests corresponding to the CTH intervention effect on the outcomes in the PP population.
- Table 5 series: This series of tables will include the results of the hypothesis tests corresponding to modification of the CTH intervention effect on the primary outcomes by pre-specified stratification variables in the ITT population.
    - Table 5a. Subgroup Analyses of Providers with a DATA 2000 Waiver During the Evaluation Period using the Intention-to-Treat Population
    - Table 5b. Subgroup Analyses of Providers with a DATA 2000 Waiver with a 30-Patient Limit During the Evaluation Period using the Intention-to-Treat Population
    - Table 5c. Subgroup Analyses of Providers with a DATA 2000 Waiver with a 100-Patient Limit During the Evaluation Period using the Intention-to-Treat Population
    - Table 5d. Subgroup Analyses of Providers with a DATA 2000 Waiver with a 275-Patient Limit During the Evaluation Period using the Intention-to-Treat Population
    - Table 5e. Subgroup Analyses of Providers with a DATA 2000 Waiver who Actively Prescribe Buprenorphine Products that are FDA approved for OUD to State Residents During the Evaluation Period using the Intention-to-Treat Population
- Table 6. Implemented Strategies from ORCCAT Menus 1-3 by Study Site for N=33 Wave 1 Communities Participating in the HEALing Communities Study

- Summary statistics of the 3 menus can be included in this table. Choice of menus to include can vary by manuscript. For this manuscript, Menu 2 will be summarized.
  - Menu 1: Overdose Education and Naloxone Distribution (OEND)
  - Menu 2: Medication for Opioid Use Disorder (MOUD)
  - Menu 3: Safer Prescribing

Note that if references to the following figures or tables are needed in a manuscript, MP0048 will be referenced to avoid reproducing the same figures or tables in MP0055.

- CONSORT Diagram
- Baseline Characteristics of Communities
- Analysis Populations, Intervention Compliance/Reasons for Noncompliance
- Safety Outcomes by Trial Arms
- Summary of Protocol Deviations by Research Site

6 REFERENCES

1. HEALing Communities Study Consortium. The HEALing (Helping to End Addiction Long-term SM) Communities Study: Protocol for a cluster randomized trial at the community level to reduce opioid overdose deaths through implementation of an integrated set of evidence-based practices. Drug Alcohol Depend. 2020 Dec 1;217:108335.
2. Moulton LH. Covariate-based constrained randomization of group-randomized trials. Clinical Trials. 2004; 1:297-305.
3. Benjamini, Y. and Hochberg, Y. Controlling the False Discovery Rate: A Practical and Powerful Approach to Multiple Testing. Journal of the Royal Statistical Society: Series B (Methodological), 1995; 57: 289-300.
4. Westgate PM, Cheng DM, Feaster DJ, Fernández S, Shoben AB, Vandergrift N. Marginal modeling in community randomized trials with rare events: Utilization of the negative binomial regression model. Clinical Trials. 2022 Apr;19(2):162-171.
5. Mancl, L. A. and DeRouen, T. A. (2001). A covariance estimator for GEE with improved small-sample properties. Biometrics 57, 126–134.
6. Kauermann, G. and Carroll, R. J. (2001). A note on the efficiency of sandwich covariance matrix estimation. Journal of the American Statistical Association 96, 1387–1396.
7. Rubin, D.B. (1987). Multiple Imputation for Nonresponse in Surveys. John Wiley & Sons Inc., New York.
8. SAS/STAT® 15.2 User's Guide

   PROC MI: https://documentation.sas.com/doc/en/statug/15.2/statug_mi_toc.htm

   PROC MIANALYZE: https://documentation.sas.com/doc/en/statug/15.2/statug_mianalyze_toc.htm

9. Allison, P. (2012). "Why You Probably Need More Imputations Than You Think." Accessed February 20, 2015. http://www.statisticalhorizons.com/more-imputations

7 APPENDICES

7.1 Appendix 1: Study Randomization

Table A1.1: HCS Wave 1 Communities

Wave	Research Site	CID	Community Name	Rural/Urban
1	KY	02	BOYD	Urban
1	KY	03	BOYLE	Rural
1	KY	06	CLARK	Urban
1	KY	07	FAYETTE	Urban
1	KY	08	FLOYD	Rural
1	KY	09	FRANKLIN	Rural
1	KY	13	KENTON	Urban
1	KY	15	MADISON	Rural
1	MA	18	BROCKTON	Urban
1	MA	19	PLYMOUTH	Urban
1	MA	20	GLOUCESTER	Urban
1	MA	22	SALEM	Urban
1	MA	23	HOLYOKE	Urban
1	MA	25	LOWELL	Urban
1	MA	28	BARNSTABLE (BOURNE/SANDWICH)	Rural
1	MA	32	MIDDLESEX (SHIRLEY/TOWNSEND)	Rural
1	NY	34	CAYUGA	Rural
1	NY	36	COLUMBIA	Rural
1	NY	38	ERIE	Urban
1	NY	40	GREENE	Rural
1	NY	41	LEWIS	Rural
1	NY	44	PUTNAM	Urban
1	NY	45	SUFFOLK	Urban
1	NY	47	ULSTER	Urban
1	OH	50	ASHTABULA	Rural
1	OH	51	ATHENS	Rural
1	OH	53	CUYAHOGA	Urban
1	OH	54	DARKE	Rural
1	OH	56	GREENE	Urban
1	OH	57	GUERNSEY	Rural
1	OH	58	HAMILTON	Urban
1	OH	61	LUCAS	Urban
1	OH	62	MORROW	Urban
1	OH	64	SCIOTO	Rural

Table A1.2: HCS Wave 2 Communities

Wave	Research Site	CID	Community Name	Rural/Urban
2	KY	01	BOURBON	Urban
2	KY	04	CAMPBELL	Urban
2	KY	05	CARTER	Rural
2	KY	10	GREENUP	Urban
2	KY	11	JEFFERSON	Urban
2	KY	12	JESSAMINE	Urban
2	KY	14	KNOX	Rural
2	KY	16	MASON	Rural
2	MA	17	NORTH ADAMS	Urban
2	MA	21	LAWRENCE	Urban
2	MA	24	SPRINGFIELD	Urban
2	MA	26	PITTSFIELD	Urban
2	MA	27	WEYMOUTH	Urban
2	MA	29	BRISTOL (BERKELEY/DIGHTON/FREETOWN)	Rural
2	MA	30	FRANKLIN (GREENFIELD/MONTAGUE/ATHOL/ORANGE)	Rural
2	MA	31	HAMPSHIRE (BELCHERTOWN/WARE)	Rural
2	NY	33	BROOME	Urban
2	NY	35	CHAUTAUQUA	Rural
2	NY	37	CORTLAND	Rural
2	NY	39	GENESEE	Rural
2	NY	42	MONROE	Urban
2	NY	43	ORANGE	Urban
2	NY	46	SULLIVAN	Rural
2	NY	48	YATES	Urban
2	OH	49	ALLEN	Urban
2	OH	52	BROWN	Urban
2	OH	55	FRANKLIN	Urban
2	OH	59	HURON	Rural
2	OH	60	JEFFERSON	Urban
2	OH	63	ROSS	Rural
2	OH	65	STARK	Urban
2	OH	66	WILLIAMS	Rural
2	OH	67	WYANDOT	Rural
