# Supplementary material for: Impact of the Communities That HEAL Intervention on Buprenorphine-Waivered Practitioners and Buprenorphine Prescribing: A Prespecified Secondary Analysis of the HCS Randomized Clinical Trial
Source: JAMA Netw Open. 2024 Feb 22;7(2):e240132. doi: 10.1001/jamanetworkopen.2024.0132 (PMC10884876; doi:10.1001/jamanetworkopen.2024.0132)
Supplement: Supplement 3. — eFigure. Raw Rate of DATA 2000 Waivered Providers and Waivered Providers Who Actively Prescribed Buprenorphine by Patient Level in Intervention and Wait-List Control Communities During Baseline and Comparison Periods eTable 1. HEALing Communities Study Sites and Underlying Population Sizes by State and Urban/Rural Classification in the Intention-to-Treat Population During the Comparison Period eTable 2. Overall Number, Rate, and Relative Rate Between Intervention and Wait-List Control Communities of Providers With a DATA 2000 Waiver in Intervention and Wait-List Control Communities by State, Urban/Rural Classification, and Patient Limit in the Intention-to-Treat Population During the Comparison Period eTable 3. Overall Number, Rate, and Relative Rate Between Intervention and Wait-List Control Communities of Providers With a DATA 2000 Who Actively Prescribe Buprenorphine for OUD to State Residents by State, Urban/Rural Classification, and Patient Limit in the Intention-to-Treat Population During the Comparison Period [file jamanetwopen-e240132-s003.pdf]

Supplementary Online Content

Stopka TJ, Babineau DC, Gibson EB, et al. Impact of the Communities That HEAL Intervention on buprenorphine-waivered practitioners and buprenorphine prescribing: a prespecified secondary analysis of the HCS randomized clinical trial. *JAMA Netw Open*. 2024;7(2):e240132. doi:10.1001/jamanetworkopen.2024.0132

eFigure. Raw Rate of DATA 2000 Waivered Providers and Waivered Providers Who Actively Prescribed Buprenorphine by Patient Level in Intervention and Wait-List Control Communities During Baseline and Comparison Periods

eTable 1. HEALing Communities Study Sites and Underlying Population Sizes by State and Urban/Rural Classification in the Intention-to-Treat Population During the Comparison Period

eTable 2. Overall Number, Rate, and Relative Rate Between Intervention and Wait-List Control Communities of Providers With a DATA 2000 Waiver in Intervention and Wait-List Control Communities by State, Urban/Rural Classification, and Patient Limit in the Intention-to-Treat Population During the Comparison Period

eTable 3. Overall Number, Rate, and Relative Rate Between Intervention and Wait-List Control Communities of Providers With a DATA 2000 Who Actively Prescribe Buprenorphine for OUD to State Residents by State, Urban/Rural Classification, and Patient Limit in the Intention-to-Treat Population During the Comparison Period

This supplementary material has been provided by the authors to give readers additional information about their work.

© 2024 Stopka TJ et al. *JAMA Network Open.*

eFigure. Raw rate of DATA 2000 waivered providers and waivered providers who actively prescribed buprenorphine by patient level in intervention and wait-list control communities during baseline and comparison periods

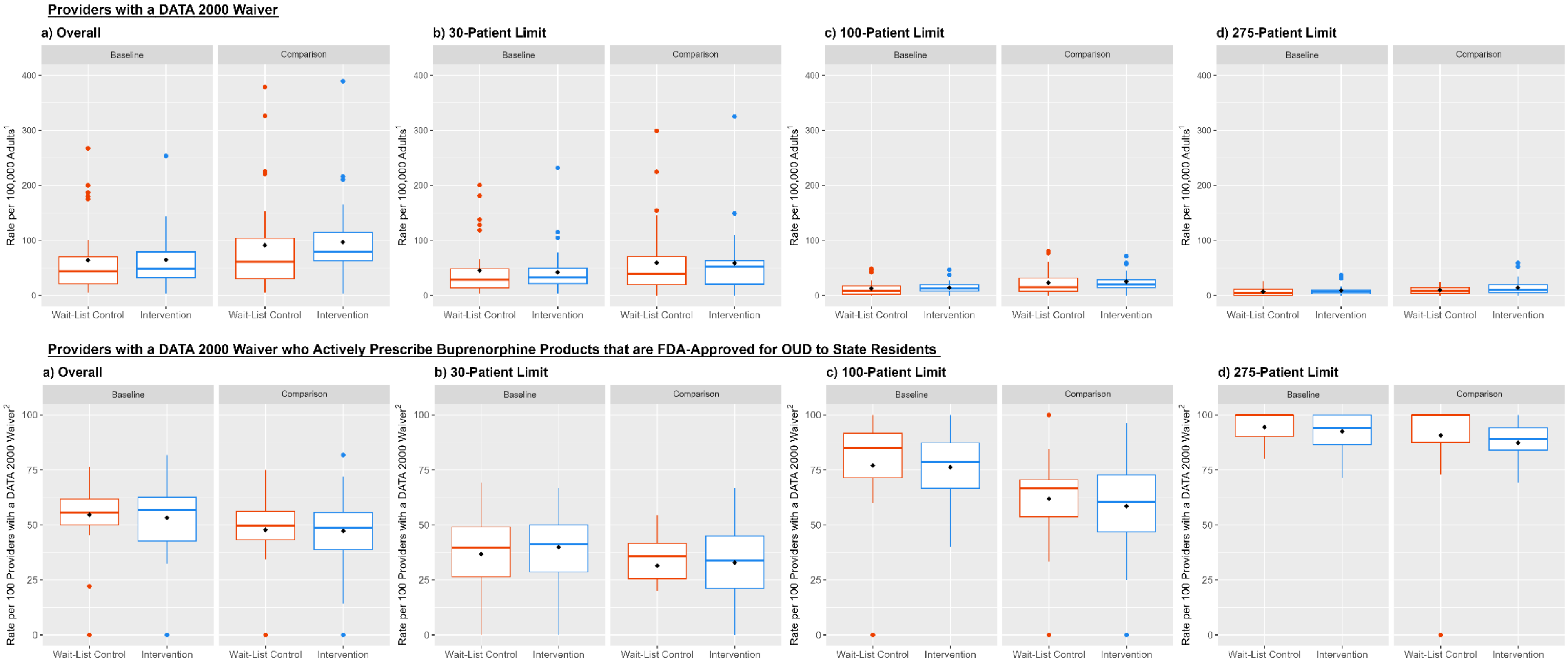

[1] Rate within a community is calculated using the ratio of the number of providers with a DATA 2000 waiver (overall or with a 30, 100, or 275-patient limit) within a community to the number of adults within a community multiplied by 100,000.

[2] Rate within a community is calculated using the ratio of the number of providers with a DATA 2000 waiver (overall or with a 30, 100, or 275-patient limit) who actively prescribe buprenorphine products that are FDA-approved for OUD to state residents within a community to the number of providers with a DATA 2000 waiver (overall or with a 30, 100, or 275-patient limit respectively) within a community multiplied by 100.

© 2024 Stopka TJ et al. *JAMA Network Open.*

eTable 1. HEALing Communities Study sites and underlying population sizes by state and urban/rural classification in the intention-to-treat population during the comparison period

Group	Intervention		Wait-List Control	
	Communities, No.[1]	Population Size, Mean (SD)[2]	Communities, No.[1]	Population Size, Mean (SD)[2]
**Overall**	34	130,563.8 (200,088.0)	33	114,313.2 (201,417.3)
**State**				
KY	8	77,230.1 (80,938.9)	8	101,970.5 (202,045.3)
MA	8	44,914.3 (26,559.3)	8	44,568.1 (33,628.8)
NY	8	137,687.1 (140,779.9)	8	122,008.6 (106,012.1)
OH	10	236,051.8 (322,922.0)	9	180,439.8 (325,173.2)
**Urban/Rural Classification**				
Urban	19	199,650.2 (248,385.2)	19	170,666.5 (252,614.9)
Rural	15	43,054.5 (19,075.4)	14	37,833.8 (23,733.0)

[1]Number of communities randomized in the specified group that are included.

[2]Number of individuals 18 years of age or older in the specified group during the comparison period. For communities that represent counties (n=48 of 67), population estimates are from 2020 Bridged-Race Population Estimates retrieved via https://www.cdc.gov/nchs/nvss/bridged_race.htm. For communities that represent units smaller than counties (n=19 of 67), population estimates are from 2017-2021 American Community Survey 5-Year Estimates retrieved via https://data.census.gov/cedsci.

© 2024 Stopka TJ et al. *JAMA Network Open.*

eTable 2. Overall number, rate, and relative rate between intervention and wait-list control communities of providers with a DATA 2000 waiver in intervention and wait-list control communities by state, urban/rural classification, and patient limit in the intention-to-treat population during the comparison period

Outcome	Group	Intervention				Wait-List Control				Overall Relative Rate[5]
		Communities, No. (%)[1]	Overall Number of Providers with a DATA 2000 waiver, No.[2]	Overall Population Size, No.[3]	Overall Rate[4]	Communities, No. (%)[1]	Overall Number of Providers with a DATA 2000 waiver, No.[2]	Overall Population Size, No.[3]	Overall Rate[4]	
Number of Providers with a DATA 2000 Waiver	**Overall**	34 (100.0)	4,177	4,439,170	94.1	33 (100.0)	3,572	3,772,336	94.7	0.99
	**State**									
	KY	8 (100.0)	723	617,841	117	8 (100.0)	485	815,764	59.5	1.97
	MA	8 (100.0)	554	359,314	154.2	8 (100.0)	816	356,545	228.9	0.67
	NY	8 (100.0)	905	1,101,497	82.2	8 (100.0)	785	976,069	80.4	1.02
	OH	10 (100.0)	1,995	2,360,518	84.5	9 (100.0)	1,486	1,623,958	91.5	0.92
	**Urban/Rural Classification**									
	Urban	19 (100.0)	3,707	3,793,353	97.7	19 (100.0)	3,169	3,242,663	97.7	1
	Rural	15 (100.0)	470	645,817	72.8	14 (100.0)	403	529,673	76.1	0.96
Number of Providers with a DATA 2000 Waiver with a 30-Patient Limit	**Overall**	34 (100.0)	2,756	4,439,170	62.1	33 (100.0)	2,369	3,772,336	62.8	0.99
	**State**									
	KY	8 (100.0)	361	617,841	58.4	8 (100.0)	279	815,764	34.2	1.71
	MA	8 (100.0)	379	359,314	105.5	8 (100.0)	559	356,545	156.8	0.67
	NY	8 (100.0)	635	1,101,497	57.6	8 (100.0)	531	976,069	54.4	1.06
	OH	10 (100.0)	1,381	2,360,518	58.5	9 (100.0)	1,000	1,623,958	61.6	0.95
	**Urban/Rural Classification**									
	Urban	19 (100.0)	2,492	3,793,353	65.7	19 (100.0)	2,121	3,242,663	65.4	1
	Rural	15 (100.0)	264	645,817	40.9	14 (100.0)	248	529,673	46.8	0.87
Number of Providers with a	**Overall**	34 (100.0)	953	4,439,170	21.5	33 (100.0)	811	3,772,336	21.5	1
	**State**									
	KY	8 (100.0)	194	617,841	31.4	8 (100.0)	127	815,764	15.6	2.02

© 2024 Stopka TJ et al. *JAMA Network Open.*

Outcome	Group	Intervention				Wait-List Control				Overall Relative Rate[5]
		**Communities, No. (%)[1]**	**Overall Number of Providers with a DATA 2000 waiver, No.[2]**	**Overall Population Size, No.[3]**	**Overall Rate[4]**	**Communities, No. (%)[1]**	**Overall Number of Providers with a DATA 2000 waiver, No.[2]**	**Overall Population Size, No.[3]**	**Overall Rate[4]**	
DATA 2000 Waiver with a 100-Patient Limit	MA	8 (100.0)	134	359,314	37.3	8 (100.0)	193	356,545	54.1	0.69
	NY	8 (100.0)	200	1,101,497	18.2	8 (100.0)	181	976,069	18.5	0.98
	OH	10 (100.0)	425	2,360,518	18	9 (100.0)	310	1,623,958	19.1	0.94
	**Urban/Rural Classification**									
	Urban	19 (100.0)	830	3,793,353	21.9	19 (100.0)	710	3,242,663	21.9	1
	Rural	15 (100.0)	123	645,817	19	14 (100.0)	101	529,673	19.1	1
Number of Providers with a DATA 2000 Waiver with a 275-Patient Limit	**Overall**	34 (100.0)	468	4,439,170	10.5	33 (100.0)	392	3,772,336	10.4	1.01
	**State**									
	KY	8 (100.0)	168	617,841	27.2	8 (100.0)	79	815,764	9.7	2.81
	MA	8 (100.0)	41	359,314	11.4	8 (100.0)	64	356,545	18	0.64
	NY	8 (100.0)	70	1,101,497	6.4	8 (100.0)	73	976,069	7.5	0.85
	OH	10 (100.0)	189	2,360,518	8	9 (100.0)	176	1,623,958	10.8	0.74
	**Urban/Rural Classification**									
	Urban	19 (100.0)	385	3,793,353	10.1	19 (100.0)	338	3,242,663	10.4	0.97
	Rural	15 (100.0)	83	645,817	12.9	14 (100.0)	54	529,673	10.2	1.26

[1]Number of communities (% randomized) in the specified group that are included. Communities in the specified group that have suppressed event and/or population data are not included.
[2]Sum of the number of providers with a DATA 2000 waiver in the specified group across all communities during the comparison period.
[3]Sum of the number of individuals 18 years of age or older in the specified group across all communities during the comparison period. For communities that represent counties (n=48 of 67), population estimates are from 2020 Bridged-Race Population Estimates retrieved via https://www.cdc.gov/nchs/nvss/bridged_race.htm. For communities that represent units smaller than counties (n=19 of 67), population estimates are from 2017-2021 American Community Survey 5-Year Estimates retrieved via https://data.census.gov/cedsci.
[4]Overall rate is calculated as the ratio of the sum of the number of DATA 2000 waivers in the specified group across all communities during the comparison period to the sum of the number of individuals 18 years of age or older in the specified group across all communities during the comparison period multiplied by 100,000.
[5]Overall relative rate is calculated as the ratio of the overall rate of providers with a DATA 2000 waiver in the specified intervention group during the comparison period to the overall rate of the providers with a DATA 2000 waiver in the specified wait-list group during the comparison period.

© 2024 Stopka TJ et al. *JAMA Network Open.*

eTable 3. Overall number, rate, and relative rate between intervention and wait-list control communities of providers with a DATA 2000 waiver who actively prescribe buprenorphine for OUD to state residents by state, urban/rural classification, and patient limit in the intention-to-treat population during the comparison period

Group	Intervention				Wait-List Control				Overall Relative Rate[5]
	Communities, No. (%)[1]	Overall number of Providers with a DATA 2000 Waiver Actively Prescribing Buprenorphine, No.[2]	Overall Number of Providers with a DATA 2000 Waiver, No.[3]	Overall Rate[4]	Communities, No. (%)[1]	Overall number of Providers with a DATA 2000 Waiver Actively Prescribing Buprenorphine, No.[2]	Overall Number of Providers with a DATA 2000 Waiver, No.[3]	Overall Rate[4]	
**Overall**	33 (97.1)	1,918	4,176	45.9	30 (90.9)	1,660	3,549	46.8	0.98
**State**									
KY	8 (100.0)	359	723	49.7	8 (100.0)	190	485	39.2	1.27
MA	8 (100.0)	247	554	44.6	7 (87.5)	393	815	48.2	0.92
NY	8 (100.0)	478	905	52.8	8 (100.0)	415	785	52.9	1
OH	9 (90.0)	834	1,994	41.8	7 (77.8)	662	1,464	45.2	0.92
**Urban/Rural Classification**									
Urban	18 (94.7)	1,692	3,706	45.7	18 (94.7)	1,456	3,155	46.1	0.99
Rural	15 (100.0)	226	470	48.1	12 (85.7)	204	394	51.8	0.93
**Patient Limit**									
30	27 (79.4)	927	2,690	34.5	27 (81.8)	793	2,339	33.9	1.02
100	28 (82.4)	562	928	60.6	24 (72.7)	514	795	64.7	0.94
275	24 (70.6)	379	446	85.0	25 (75.8)	331	383	86.4	0.98

[1]Number of communities (% randomized) in the specified group that are included. Communities in the specified group that have suppressed event and/or population data are not included.
[2]Sum of the number of providers with a DATA 2000 waiver who actively prescribe buprenorphine for OUD to state residents in the specified group across all communities during the comparison period.
[3]Sum of the number of providers with a DATA 2000 waiver in the specified group across all communities during the comparison period.
[4]Overall rate is calculated as the ratio of the sum of the number of providers with a DATA 2000 waiver who actively prescribe buprenorphine for OUD to state residents in the specified group across all communities during the comparison period to the sum of the number of providers with a DATA 2000 waiver in the specified group across all communities during the comparison period multiplied by 100.
[5]Overal relative rate is calculated as the ratio of the overall rate of providers with a DATA 2000 waiver who actively prescribe buprenorphine for OUD to state residents in the specified intervention group during the comparison period to the overall rate of the providers with a DATA 2000 waiver who actively prescribe buprenorphine for OUD to state residents in the specified wait-list group during the comparison period.

© 2024 Stopka TJ et al. *JAMA Network Open.*
