# Supplementary material for: Impact of the Communities That HEAL Intervention on Buprenorphine-Waivered Practitioners and Buprenorphine Prescribing: A Prespecified Secondary Analysis of the HCS Randomized Clinical Trial
Source: JAMA Netw Open. 2024 Feb 22;7(2):e240132. doi: 10.1001/jamanetworkopen.2024.0132 (PMC10884876; doi:10.1001/jamanetworkopen.2024.0132)
Supplement: Supplement 4. — Data Sharing Statement [file jamanetwopen-e240132-s004.pdf]

Data Sharing Statement

Stopka. Impact of the Communities That HEAL Intervention on Buprenorphine-Waivered Practitioners and Buprenorphine Prescribing. *JAMA Netw Open*. Published February 22, 2024. doi:10.1001/jamanetworkopen.2024.0132

Data
Data available: Yes
Data types: Deidentified participant data, Data dictionary
How to access data: University of Michigan's Inter-university Consortium for Political and Social Research (ICPSR) will archive HCS data. ICPSR home page: https://www.icpsr.umich.edu/web/pages/
When available: beginning date: 03-31-2025

Supporting Documents
Document types: None

Additional Information
Who can access the data: Researchers whose proposed use of the data has been approved
Types of analyses: For any purpose
Mechanisms of data availability: With a signed data access agreement
